# Hormone priming and metabolic engineering of phytohormone crosstalk in rice under combined biotic and abiotic stresses: a multi-omics perspective for climate-resilient crop development

**DOI:** 10.3389/fpls.2026.1924558

**Published:** 2026-08-19

**Authors:** Zhu Shuixing, Zhu Jing, Dikhnah Alshehri, Hadba Al-Amrah, Irfan Manzoor, Yu Aiying

**Affiliations:** 1 Xianju Agricultural Technology Extension Center, Xianju, Zhejiang, China; 2 Department of Biology, Faculty of Science, University of Tabuk, Tabuk, Saudi Arabia; 3 Department of Biological Sciences, Faculty of Science, King Abdulaziz University, Jeddah, Saudi Arabia; 4 Department of Bioinformatics and Biotechnology, Government College University Faisalabad (GCUF), Faisalabad, Pakistan

**Keywords:** climate-resilient breeding, CRISPR, hormone priming, multi-omics, *Oryza sativa*, phytohormone crosstalk

## Abstract

Rice (*Oryza sativa* L.) is the caloric backbone for more than half of humanity, yet it remains one of the most vulnerable crops to the simultaneous biotic and abiotic stresses exacerbated by climate change. Phytohormone priming and the complex crosstalk networks governed by transcription factor hubs like *WRKY*, *MYB*, and *NAC* serve as the central adaptive mechanism for stress resilience. This review synthesizes how multi-omics integration, including spatial and single-cell transcriptomics, is resolving the molecular architecture of hormonal priming and epigenetic stress memory. We critically evaluate advanced metabolic engineering and genome-editing strategies such as CRISPR-Cas9, base/prime editing, and synthetic gene circuits that enable precision modifications to decouple stress tolerance from historical yield penalties. Furthermore, we discuss the emerging roles of microbiome-assisted priming via synthetic consortia and the application of artificial intelligence and digital twins (continuously updated computational models of crop physiology) for predictive stress management. By integrating these diverse technological pillars, we propose a systems-level roadmap for developing climate-resilient rice cultivars capable of maintaining yield stability across a volatile combinatorial stress landscape. This synthesis provides a framework for translating mechanistic hormonal insights into field-applicable cultivars to ensure global food security.

## 1 Introduction

Rice (*Oryza sativa* L.) constitutes the primary caloric source for more than half of humanity, and together China and India alone account for over half of global production, with India supplying roughly 40% of the rice traded internationally (Mohidem et al., 2022). That concentration is precisely what makes the vulnerability of rice to climate change a food-security issue rather than a regional agronomic concern: a poor season in a handful of producing countries reverberates through global commodity prices within months (Abeysekara and Rathnayake, 2024).

The relationship between climate change and rice yield is more nuanced than a simple decline narrative (Algarni et al., 2025). A recent analysis combining five decades of observational and process-based modeling data found that global rice production nearly doubled between the 1960s and the 2010s, driven primarily by expanded irrigation, increased nitrogen inputs, and the fertilization effect of rising atmospheric CO_2_ but the same study isolated climate change as the only factor that actively suppressed yields, cutting global rice production by an estimated 7% between 2006 and 2015 through warming, heat stress, and water shortages, based on a global-scale process-based modeling analysis (Lin and Jain, 2026). In other words, agronomic management has so far been masking, not reversing, a real and growing climate penalty.

The yield consequences of this volatility are already substantial. Across crops broadly, drought alone removes an estimated 10-30% of annual yield, while pests and diseases account for a further 20-40% in losses (Tao and Chen, 2025). In rice specifically, the effects are severe: drought can cause up to 40% production loss in Asia, and bacterial leaf blight can remove up to 74% of yield in tropical Asia. Bacterial panicle blight is capable of cutting both yield and milling quality by up to 75% where it establishes (Malik et al., 2026).

Most of what is known about rice stress physiology comes from single-stress experiments conducted in isolation under controlled conditions. Field conditions expose rice plants to simultaneous stress combinations that cannot be adequately represented by single-stress experimental systems (Suzuki et al., 2014), in what remains the field’s most-cited framing of the problem, demonstrated that plant responses to combined stresses cannot be predicted by summing the individual responses combined stress generates its own distinct transcriptional and physiological program, frequently dominated by antagonistic signaling that leaves the plant more vulnerable than either stress alone would suggest.

Rice-specific evidence bears this out directly. When (Pal et al., 2022) exposed two contrasting genotypes to combined drought and *Xanthomonas oryzae* pv. oryzae (Xoo) infection, even the resistant genotype showed higher lesion severity under combined stress than under pathogen challenge alone, and TN1 supported up to two-fold higher pathogen multiplication. Under combined salinity and pathogen pressure, plants tend to prioritize ionic homeostasis activating *NHX* and SOS pathways at the expense of pathogenesis-related protein synthesis that pathogen defense requires (Sackey et al., 2025).

By contrast, heat pre-treatment actually enhanced resistance to brown planthopper (BPH, *Nilaparvata lugens*) in a one-directional cross-protection effect traced to shared regulatory genes, demonstrating that not all combined stresses produce antagonistic outcomes (Cao et al., 2025). These findings collectively explain why single-stress studies are insufficient as a basis for cultivar development, and why hormone priming strategies targeting multiple pathways simultaneously are needed.

Priming, pre-exposing a plant to a mild elicitor so that its response to a subsequent, more severe stress is faster and stronger, has moved from a descriptive concept to a mechanistically tractable target. A 2025 update on phytohormone-based priming documents that ABA, JA, SA, and GA3 priming consistently improves subsequent stress outcomes, while cataloguing practical delivery methods now including nanoparticle-based systems that improve uptake efficiency (Matkowski and Daszkowska–Golec, 2025). CRISPR/Cas9 has simultaneously become the widely adopted tool for stably engineering hormone-pathway components in rice, with precision editing of ABA receptor genes, JAZ repressors, and SA catabolism enzymes producing measurable multi-stress tolerance gains (Malik et al., 2026).

**Figure 1:**
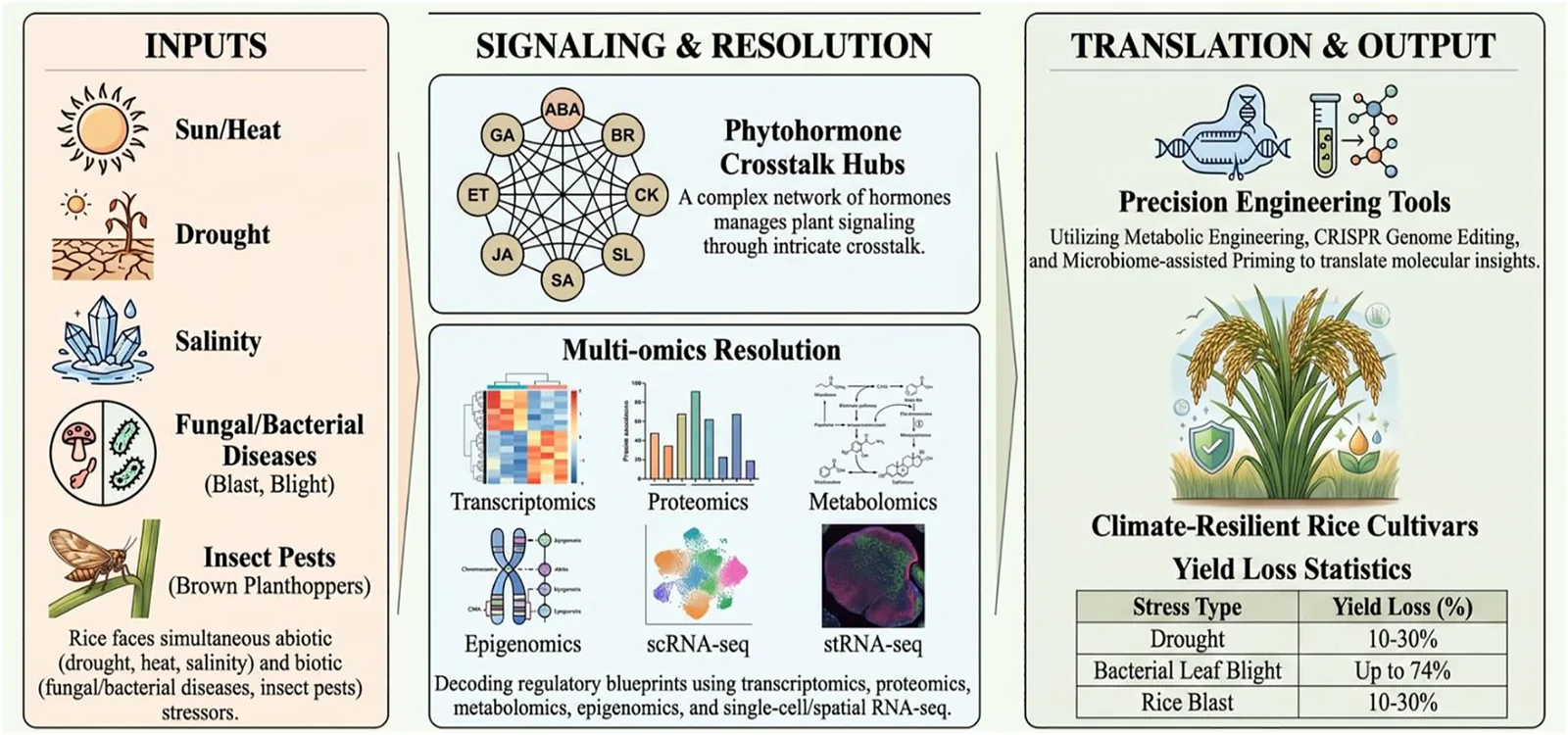
Conceptual framework for climate-resilient rice development. Rice in agricultural environments is simultaneously exposed to multiple biotic and abiotic stresses (left). These diverse signals are integrated through phytohormone crosstalk hubs and resolved at high resolution by multi-omics platforms, including transcriptomics, proteomics, metabolomics, epigenomics, and single-cell/spatial transcriptomics (center). These mechanistic insights are translated into climate-resilient rice cultivars through three primary technological pillars: metabolic engineering, CRISPR genome editing, and microbiome-assisted priming (right). ABA, abscisic acid; JA, jasmonic acid; SA, salicylic acid; ET, ethylene; GA, gibberellin; BR, brassinosteroid; SL, strigolactone; CK, cytokinin; scRNA-seq, single-cell RNA sequencing; stRNA-seq, spatial transcriptomic RNA sequencing.

This review synthesizes current understanding of phytohormone priming and crosstalk in rice under combined biotic and abiotic stress. We focus on the nine major phytohormone classes and the crosstalk networks connecting them, evaluate how multi-omics platforms are resolving the molecular architecture of priming, and assess metabolic engineering and genome-editing strategies translating mechanistic insights into deployable traits. Our objective is to identify where the field’s mechanistic knowledge has outpaced its field-validated applications and to propose systems-level and precision-breeding priorities needed to close that gap for climate-resilient rice cultivar development (**Figure 1**).

Note on Literature Coverage: This manuscript is a narrative review with systematic literature coverage and does not require a formal PRISMA flowchart. Literature was identified through structured searches of PubMed, Web of Science, Scopus, and Google Scholar using Boolean search terms combining (“rice” OR “*Oryza sativa*”) AND (“phytohormone priming” OR “hormone priming” OR “ABA” OR “jasmonic acid” OR “salicylic acid” OR “CRISPR” OR “multi-omics”) AND (“combined stress” OR “biotic stress” OR “abiotic stress” OR “genome editing”). The search period spanned 2000 to July 2026, with emphasis on publications from 2019 to 2026. Inclusion was limited to peer-reviewed primary research articles and authoritative reviews published in English-language journals; preprints and non-peer-reviewed sources were excluded. Where multiple studies reported conflicting findings, both perspectives are presented and the basis of disagreement discussed. Reference selection prioritized studies with multi-environment, multi-genotype, or field-validated data over single-condition laboratory reports, consistent with standard practice in narrative reviews.

## 2 Environmental stresses and their impact on rice productivity

### 2.1 Abiotic stresses

#### 2.1.1 Drought

Among the abiotic constraints of rice, drought is the most extensively characterized and arguably the most damaging, capable of removing 50-70% of yield in severe cases and affecting an estimated 55 million people’s livelihoods globally each year (Ma et al., 2024). The current mechanistic picture centers on ABA: rice perceives water deficit through hydraulic and chemical signals and drives stomatal closure and osmotic adjustment via ABA-dependent transcriptional reprogramming through MAPK and Ca^2+^ cascades. Drought tolerance and grain yield have historically been antagonistic breeding targets, a tension that recent gene-editing work (Section 8) is now starting to decouple. Transcription factor families *DREB*, *NAC*, and *bZIP* act as the major regulatory hubs (Li et al., 2024b).

#### 2.1.2 Salinity

Salinity stress operates through ionic toxicity from Na^+^ accumulation and osmotic stress from reduced water potential both converging on oxidative damage. Salinity-affected soils now account for roughly 20% of arable land and nearly half of irrigated land worldwide (Sackey et al., 2025). Rice manages ionic stress primarily through coordinated *SOS* and *NHX* pathway activity. This ionic-defense priority comes at a measurable cost to pathogen defense when the two stresses co-occur (Section 2.3).

#### 2.1.3 Heat

Heat stress is the most climate-sensitive vulnerability in rice because it strikes hardest during the reproductive window: pollen viability collapses, anther dehiscence is impaired, and spikelet sterility follows directly, with empirical data showing that each 1 °C rise in temperature corresponds to roughly a 3.85% yield reduction (Zhang et al., 2025). Mechanistically, heat disrupts membrane lipid homeostasis, destabilizes the photosynthetic apparatus, and triggers a regulatory cascade spanning heat-shock transcription factors, RNA stability control, epigenetic remodeling, and hormone signaling (Kumar et al., 2026).

#### 2.1.4 Cold

Cold stress constrains rice at the opposite climatic extreme, limiting both the geographic range and yield stability. The cold-sensor complex *COLD1-RGA1* and the more recently discovered *COG1-OsSERL2* complex both trigger Ca^2+^ and 2’,3’-cAMP secondary-messenger signaling that activates the core *OsbHLH002*/*OsICE1-OsDREBs*-COR transcriptional module (Xiao et al., 2026). A landmark 2024 discovery identified a previously unknown *COLD6-OSM1* signaling module suggesting core cold-sensing mechanisms are still being discovered (Shahzad et al., 2024).

#### 2.1.5 Flooding

Flooding is mechanistically distinct because its primary damage vector is gas-exchange restriction. Rice has evolved two opposing adaptive strategies: the ‘escape’ strategy of rapid internode elongation in deepwater rice, and the ‘quiescence’ strategy controlled by the *SUB1A* locus, which specifically restricts ethylene-induced gibberellin responsiveness (Ashikari et al., 2025). Field deployment of Sub1 varieties shows a 2–3 ton/ha yield advantage over near-isogenic non-Sub1 lines following a submergence event.

### 2.2 Biotic stresses

The major biotic threats in rice span bacterial, fungal, and viral pathogens as well as insect pests, each intersecting with the phytohormone network in distinct ways. Bacterial blight (BB), caused by *Xanthomonas oryzae* pv. oryzae, and bacterial leaf streak (BLS), caused by *X. oryzae* pv. oryzicola, are the two dominant bacterial threats in rice, with BB capable of yield losses exceeding 50% and reaching as much as 70-75% under the severe kresek syndrome, while BLS causes 8-32% losses (Qi et al., 2025). Resistance operates through pattern-triggered immunity and effector-triggered immunity, with the pathogen deploying TALE effectors that hijack host SWEET-family sugar transporters.

Rice blast, caused by *Magnaporthe oryzae*, destroys an estimated 10-30% of global rice production each year enough rice to feed roughly 60 million people at an estimated cost of $66 billion (Oliveira‐Garcia et al., 2024). The fungus infects through a specialized appressorium structure and deploys effector proteins through a biotrophic interfacial complex that suppresses rice immune signaling from within living host cells.

Viral diseases also impose significant losses: rice tungro disease (RTD), caused by the joint infection of RTBV and RTSV and transmitted by leafhopper vectors, produces an estimated $109 million in annual global losses and reduces national production in India by roughly 2% on average (Dey et al., 2024). Because RTD requires co-infection by two unrelated viruses, vector control of the leafhopper is often as important to disease management as host resistance itself.

Among insect pests, stem borers alone account for an estimated 40% of total pest-induced yield loss in rice, with the yellow stem borer (*Scirpophaga incertulas*) causing 25-70% localized yield loss depending on growth stage, while economic losses in China alone are estimated at CNY 11.5 billion annually (Cao et al., 2025). Rice resistance to insect feeding is coordinated through the same SA and JA signaling pathways central to this review, meaning insect resistance and phytohormone priming strategies are not separate research tracks (Abbas et al., 2026).

### 2.3 Combined stress effects on rice

Three specific interaction patterns recur across the rice literature and form the direct empirical foundation for this review’s phytohormone priming argument. First, resource competition between defense programs: under combined drought and bacterial blight, rice activates a shared ‘core’ stress response dominated by ABA, JA, and SA signaling regardless of which stress initiated it, meaning the two stresses draw on the same finite hormonal capacity rather than mounting independent defenses (Vemanna et al., 2019; Pal et al., 2022). Second, stress combinations can produce genuine cross-protection: heat pre-treatment measurably enhances BPH resistance through shared regulatory genes activated by both stressors (Cao et al., 2025). Third, abiotic stress can directly increase biotic vulnerability through physical channels independent of hormone crosstalk, as in submergence-induced susceptibility to pests and diseases (Ashikari et al., 2025). Taken together, these findings justify treatingphytohormone priming and crosstalk rather than single-pathway engineering as the more promising lever for combined-stress tolerance.

## 3 Phytohormone priming: concepts and mechanisms

### 3.1 Definition and types of priming

Priming describes a pre-exposure strategy in which a plant receives a mild, sub-damaging stimulus that places it in a sensitized state, primed to respond faster and more strongly when a real stress arrives later (Cañizares et al., 2025). Current classification recognizes hydropriming, osmopriming, hormonal priming (direct application of ABA, SA, JA, or GA3), bio-priming, nutrient priming, and nanoparticle priming (Jatana et al., 2024; Matkowski and Daszkowska–Golec, 2025). Understanding how these diverse priming methods converge on a common adaptive outcome requires examination of their shared molecular basis. What unites these diverse methods is that they all reach the same downstream target: the plant’s own hormonal network. For example, humic acid seed priming recalibrates the ABA-GA balance by simultaneously downregulating ABA biosynthesis genes (*OsNCED1*, *OsABA8ox2*) and upregulating GA biosynthesis genes (*OsGA3ox1*, *OsGA2ox2*) before the seed ever encounters stress (Jatana et al., 2024).

### 3.2 Molecular basis of priming

At the cellular level, priming begins with rapid signaling events increases in cytosolic Ca^2+^, a burst of reactive oxygen species, and cAMP which activate MAPK and CDPK kinase cascades (Aswathi et al., 2025). What separates a primed response from an ordinary one is the molecular ‘marks’ retained after the initial stimulus subsides: histone modifications, accumulated but inactive transcription factors, and altered small RNA populations that lower the activation threshold for the same pathway during a subsequent encounter. *WRKY, MYB,* and *NAC* transcription factor families are consistently identified as the regulatory hubs where this poised state is held.

In rice specifically, microRNAs have emerged as a major layer of the priming machinery. Thermopriming differentially regulates a defined set of microRNAs including osa-miR156b-3p, osa-miR167e-3p, osa-miR168a-5p, osa-miR531b, osa-miR1846d-5p, osa-miR5077, and osa-miR5149 whose target genes encode heat-shock proteins and transcription factors, with several mitochondrial and chloroplastic HSPs showing differential regulation specifically in thermoprimed plants (Ganie et al., 2024).

### 3.3 Stress memory and primed state

The molecular marks described above constitute what the field now calls stress memory. Rice plants primed with salt stress at the vegetative stage retain a long-term somatic memory that manifests as cross-tolerance to drought at the grain-filling stage, expressed through higher photosynthetic capacity, stronger antioxidant activity, and enhanced root growth relative to non-primed plants (Rossatto et al., 2023). This means a single, early-stage priming intervention targeting one stress can confer protection against an entirely different stress encountered weeks later.

UV-B priming reduces oxidative damage and boosts protective flavonoid and anthocyanin accumulation, and rice plants primed with UV-B before PEG-induced osmotic stress show elevated expression of the antioxidant enzymes Cu/Zn-SOD, CatA, and APX1 (Thomas and Puthur, 2020; Thomas et al., 2020). This pattern is not unique to rice in tobacco, combined UV-B exposure and exogenous H_2_O_2_ pretreatment induce cross-tolerance to subsequent drought stress via elevated catalase, peroxidase, and PAL activity (Sáenz‐de La O et al., 2021), suggesting the priming-driven interconnection of abiotic-stress pathways reflects a broader, conserved plant strategy.

### 3.4 Advantages of hormone priming over conventional approaches

Set against conventional breeding and transgenic engineering, phytohormone priming offers a distinct practical profile. Conventional breeding for stress tolerance is slowed by complex trait inheritance and multi-year selection cycles (Ganie et al., 2024). Transgenic engineering can be precise stress-induced expression of the cytokinin biosynthesis gene IPT in wheat measurably reduced the grain-yield penalty under drought while improving yield under well-watered conditions (Beznec et al., 2021) but GMO regulatory frameworks in the European Union and consumer-acceptance challenges create barriers that priming-based approaches simply do not encounter (Cañizares et al., 2025). Hormone priming requires no permanent genetic modification, works directly with the plant’s existing hormonal architecture, and is comparatively inexpensive to deploy at scale via seed treatment. Its principal limitation is that effects can be less durable and more genotype- and dose-dependent than a stably integrated transgene which is why this review treats priming and metabolic/genetic engineering as complementary strategies rather than competing ones.

## 4 Major phytohormones involved in rice stress adaptation

### 4.1 Abscisic acid

In rice, ABA is the central stress-integration hormone virtually every abiotic stress pathway converges on it through the PYL receptor family, PP2C phosphatases, and SnRK2 kinases (Ma et al., 2024). The hormone’s tolerance benefits can be decoupled from yield cost: CRISPR knockout of *OsPYL9* raises endogenous ABA and improves drought tolerance while simultaneously increasing grain yield under both drought and well-watered conditions (Usman et al., 2020), and *OsOLP1* contributes to drought tolerance through a defined ABA biosynthesis-proline-lignin axis (Yan et al., 2023). ABA’s reach extends to salinity, cold, and combined-stress signaling (**Table 1**).

**Table 1:** Comprehensive catalog of phytohormone priming approaches in rice (*Oryza sativa* L.) under biotic and abiotic stress conditions.

Hormone	Stress	Priming method	Molecular mechanism	Physiological/agronomic outcome	Reference
ABA	Drought	Seed soaking (100 µM) or foliar spray (50-100 µM)	Activation of SnRK2-ABRE cascade; upregulation of *OsNCED3*, *OsDREB, SNAC1, OsLEA*; stomatal closure via PYL-PP2C-SnRK2 module	Improved photosynthesis retention, higher survival rate;15-30% yield protection under moderate drought in experimental studies	(Ma et al., 2024)
ABA	Salinity	Foliar spray (50-100 µM) at seedling stage	Upregulation of *SOS1, NHX1, OsHKT1*; activation of antioxidant enzymes (CAT, APX, POD); reduced Na^+^ accumulation in leaves	Reduced MDA and H_2_O_2_; higher chlorophyll retention; improved ion homeostasis; genotype-dependent magnitude	(Chen et al., 2022; Sackey et al., 2025)
ABA	Cold	PEG-based osmopriming + ABA pretreatment	Activation of *OsbHLH002/OsICE1-OsDREBs*-COR module; 2-3x elevated endogenous ABA; *COLD1-RGA1* sensitized	Higher seedling survival under cold; improved germination at 13–19 degrees C	(Kumar et al., 2020; Shahzad et al., 2024)
JA/MeJA	Rice blast (M. oryzae)	Foliar spray (50-100 µM MeJA) or seed priming	COI1-JAZ-*OsMYC2* cascade; upregulation of *OsPR* genes and trypsin inhibitors (TLPs); *LOX3*-mediated JA biosynthesis induction; *OsJAZ8* repressor degradation	Restored blast resistance in *LOX3*-deficient lines;reduced lesion size by 40-60% under controlled inoculation conditions; elevated PR1/PR3/PR5 expression	(Su et al., 2023; Taniguchi et al., 2023)
JA/MeJA	Insect pests (BPH, stem borer)	Wound-induced or exogenous MeJA application	*OsMYC2/OsbHLH034/RERJ1* activation; trypsin inhibitor and TLP induction; beta-cyclocitral volatile emission regulated by *OsJAZ8*	Reduced BPH feeding damage; lower stem borer survival; indirect defense via attracting natural enemies	(Cao et al., 2025; Ge et al., 2025)
JA/MeJA	Combined drought + biotic	Exogenous ABA or MeJA; joint ABA-JA pathway activation	Reciprocal induction: ABA upregulates *OsAOS2, OsOPR, OsACOX*; JA elevates *OsNCED; WRKY/*MYC2/*bZIP* co-regulation	Simultaneous drought tolerance and BPH resistance; ABA-JA synergism active under combined stress	(Li et al., 2022; Cao et al., 2025)
SA	Bacterial blight (Xoo)	Foliar spray (0.5–1 mM SA) or BTH analog treatment	*WRKY45* activation (synergised by SA-cytokinin); *OsNPR1* stabilization; PR1/PR3/PR5 induction; H3K4me3 chromatin remodeling at defense-gene loci	Xoo lesion reduction by 30-50% in experimental systems; primed SAR activation in systemic leaves; broad-spectrum induced resistance	(Shimono et al., 2007; Tian et al., 2025)
SA	Combined salinity + pathogen	SA priming before combined salt-pathogen exposure	Maintains NPR1 stability against ABA-induced proteasomal degradation; preserves SA-JA synergism for antiviral immunity via *OsNPR1-OsMYC2* disruption	Partial restoration of pathogen resistance under salinity; limits ABA-induced susceptibility to biotrophic pathogens	(Xu et al., 2013; Meguro and Sato, 2014)
Strigolactone (GR24)	Salinity	Exogenous GR24 application (1-5 µM) at seedling stage	Enhanced SOD, APX, POD, CAT antioxidant enzyme activities; restored chloroplast ultrastructure; ABA-SL crosstalk through shared carotenoid biosynthetic precursor pool	Reduced oxidative damage; improved gas exchange parameters and photosynthetic efficiency under NaCl stress	(Duan et al., 2025)
Melatonin	Combined salt + drought	Exogenous melatonin (100-200 µM) seed or foliar treatment	Upregulation of *OsSOS, OsNHX, OsHSF, OsDREB*; cross-regulation with ABA, JA, and SA pathways; enhanced ROS scavenging via SOD, CAT, and POD	Reduced electrolyte leakage; improved membrane stability; multi-stress tolerance through multi-hormone network activation	(Khan et al., 2024)
Cytokinin	Drought	Stress-inducible IPT expression under PSARK promoter (transgenic)	Maintained carbon and nitrogen assimilation; delayed drought-induced senescence; SA-cytokinin synergism also activates *WRKY45* for SAR priming	Undisturbed grain filling under water deficit; improved yield under drought without compromising well-watered performance	(Reguera et al., 2013)
GA3	Cold + flooding	Seed priming with GA3 (50–100 ppm) combined with hydropriming	SUB1A-mediated GA-ethylene crosstalk; GA-ABA balance shift promoting growth resumption; *OsGA3ox* induction; DELLA protein modulation at transition zones	Improved germination and seedling vigor under cold/flooding; 2–3 t/ha yield advantage over non-primed Sub1 varieties	(Ashikari et al., 2025) (Wang et al., 2024)

#### 4.1.1 ABA-mediated priming in rice

Exogenous ABA priming delivered as seed soaking (100 µM), foliar spray (50-100 µM), or osmopriming is among the most extensively validated phytohormone priming approaches for rice. ABA priming activates the SnRK2-ABRE cascade, a pathway directly confirmed in rice through functional studies of *OsSnRK2* kinases (Ma et al., 2024), upregulates *OsNCED3*, *OsDREB*, *SNAC1*, and *OsLEA* genes, and induces stomatal closure through the PYL-PP2C-SnRK2 signaling module, collectively producing 15-30% yield protection under moderate drought in experimental studies. Among these responses, stomatal closure and osmotic adjustment are primary ABA-mediated adaptive responses, while upregulation of antioxidant enzymes (CAT, APX, POD) represents secondary protective mechanisms that mitigate stress-associated oxidative damage (Ma et al., 2024). Under salinity, foliar-applied ABA improves survival and growth potential by upregulating *SOS1*, *NHX1*, and *OsHKT1*, activating antioxidant enzymes (CAT, APX, POD), and reducing Na^+^ accumulation in leaf tissue, with the magnitude of improvement being genotype-dependent (Sackey et al., 2025). Under cold stress, ABA pretreatment combined with osmopriming sensitizes the *COLD1-RGA1* sensor complex and activates the *OsbHLH002*/*OsICE1-OsDREBs* transcriptional module, improving seedling survival at 13-19 °C (Kumar et al., 2020; Shahzad et al., 2024). The ABA priming legacy also operates epigenetically: ABA-primed plants show altered DNA methylation at *OsNCED* and *OsDREB* loci that persist through recovery periods, contributing to the stress-memory component of the primed state (Ganie et al., 2024). A comprehensive overview of ABA priming studies is presented in **Table 1**.

### 4.2 Jasmonic acid

JA’s defining feature in rice is the JAZ-COI1 repressor module. A CRISPR-induced frameshift in *OsJAZ10* generates a novel protein (FJ10) that confers BPH resistance without canonical JA signaling activation and without a growth penalty directly resolving the trade-off that JA-mediated defense has historically imposed (Li et al., 2024a) (**Table 1**). A separate study knocking out *OsJAZ12*, *OsJAZ13*, *OsCOI1b*, and *OsNINJA* found these JA-pathway genes also regulate ABA metabolism via *OsNCED1* and *OsABA8ox1* direct genetic evidence that JA and ABA pathways are mechanistically linked (Mao et al., 2025).

#### 4.2.1 JA-mediated priming in rice

Jasmonate priming (exogenous MeJA at 50-100 µM applied as foliar spray or seed treatment) primes rice defense against both pathogens and insect herbivores through COI1-JAZ-*OsMYC2* cascade activation and upregulation of OsPR genes, trypsin protease inhibitors, and thaumatin-like proteins (TLPs), and thaumatin-like proteins (TLPs). Under blast infection (*Magnaporthe oryzae*), exogenous JA application restores blast resistance in *LOX3*-deficient lines that have compromised endogenous JA biosynthesis, reducing lesion size and confirming that JA-pathway priming can compensate for genetic deficiencies in biosynthesis capacity (Su et al., 2023). Under BPH and stem borer challenge, MeJA treatment activates the bHLH transcription factors *OsMYC2*, *OsbHLH034*, and *RERJ1*, which positively regulate JA-mediated defense gene expression including trypsin proteinase inhibitors, terpene synthases, and volatile organic compounds (Taniguchi et al., 2023; Ge et al., 2025). The JA-responsive apocarotenoid volatile β-cyclocitral, whose accumulation is partly regulated by *OsJAZ8*, has been documented to induce resistance against *Xanthomonas oryzae* pv. oryzae (bacterial blight) and suppress ABA biosynthesis in rice, it acts as a defense signaling compound rather than a direct toxicant against insects (Taniguchi et al., 2023). Critically, transcriptome analysis confirms that ABA pretreatment upregulates *OsAOS2*, *OsOPR*, and *OsACOX* (JA-biosynthesis genes), while exogenous JA reciprocally elevates *OsNCED* expression, demonstrating bidirectional hormonal cross-priming through the ABA-JA crosstalk module described in Section 5.1 (Li et al., 2022). JA priming studies are comprehensively cataloged in **Table 1**.

### 4.3 Salicylic acid

SA’s role centers on biotrophic pathogen defense. Simultaneous knockout of three SA 5-hydroxylase genes (*OsS5H1/2/3*) blocks SA catabolism, raises endogenous SA, and confers broad-spectrum resistance to bacterial blight and rice blast simultaneously (Liu et al., 2023). ABA actively dismantles the SA immune hub at the protein level by inducing proteasome-mediated degradation of NPR1 explaining why drought-stressed rice is consistently more susceptible to biotrophic pathogens (Xu et al., 2013; Tian et al., 2025).

#### 4.3.1 SA-mediated priming in rice

Salicylic acid priming (foliar spray at 0.5–1 mM or application of the SA analog BTH) is the most well-established approach for inducing systemic acquired resistance (SAR) in rice. SA priming activates *WRKY45*, which is synergistically induced by SA-cytokinin signaling, and leads to *OsNPR1* stabilization and PR1/PR3/PR5 gene induction through NPR1-mediated chromatin remodeling into a transcriptionally active H3K4me3 state at defense-gene loci, reducing Xoo lesion length by 30-50% (in experimental studies) and conferring primed SAR activation upon subsequent pathogen encounter (Shimono et al., 2007; Tian et al., 2025). Under combined salinity and pathogen stress, SA priming partially maintains NPR1 protein stability against ABA-induced proteasomal degradation, preserving the pathogen resistance that would otherwise be lost to the ABA-SA antagonism described in Section 5.2 (Xu et al., 2013; Meguro and Sato, 2014). The SA-JA synergism documented in antiviral immunity, where *OsNPR1* activates JA signaling by disrupting the *OsJAZ-OsMYC2* complex, means SA priming simultaneously primes both SA-dependent and JA-dependent immune pathways, a dual-priming effect of particular value under combined biotic stress scenarios where SA-responsive and JA-responsive pathogens may co-present (Zhang et al., 2023a). SA priming studies in rice are cataloged in **Table 1**.

### 4.4 Other phytohormones in rice stress adaptation

Ethylene functions as a systemic modulator that conditions the interpretation of concurrent hormonal signals across other stress response pathways (Zhang et al., 2026). Under flooding, ethylene accumulation through MHZ1-OsEIL1 signaling can promote aerenchyma formation and lateral root initiation, with outcomes mediated almost entirely through crosstalk with auxin and ABA rather than through independent action. The ERF family with members including *OsERF83* (simultaneously drought- and pathogen-responsive) and *OsDERF1* (negative modulator of drought tolerance) provides the molecular interface through which ethylene coordinates multi-stress responses (Qin et al., 2024).

Auxin’s stress role in rice similarly operates largely through its crosstalk with ABA. *OsIAA20* regulates the ABA-response gene *OsRab21* and responds positively to both drought and salt stress. Auxin transport machinery is similarly stress-responsive: *OsPIN3t* overexpression enhances drought tolerance, and drought measurably depletes endogenous auxin in developing panicles a depletion that exogenous auxin application can partially reverse, improving pollen viability and seed-setting rate under stress (Hou et al., 2025).

Cytokinins are the phytohormone class most directly tied to the ‘stay-green’ stress-tolerance strategy. The landmark demonstration used stress-induced IPT expression under a senescence-associated promoter (PSARK::IPT): transgenic plants maintained undisturbed carbon and nitrogen assimilation during water-deficit stress while wild-type plants showed clear inhibition (Reguera et al., 2013). Expressing the Arabidopsis cytokinin-modifying glycosyltransferase UGT76C2 in rice improves both drought and salt tolerance by altering cytokinin pool composition (Li et al., 2020).

Gibberellins (GA) stress role is best understood through its antagonist DELLA (SLR1) and the 2025 discovery of an SLR1-*OsBURP3-OsSUS1* module that connects GA signaling directly to drought tolerance via sucrose-dependent stomatal aperture control (Hamed et al., 2025; Huang et al., 2025). GA and ABA jointly calibrate the growth-versus-drought-response balance in rice through a dedicated signaling module (Li et al., 2020).

Brassinosteroids (BR) occupy an unusually direct intersection of yield architecture and stress tolerance because the same semi-dwarf phenotype produced by reduced BR signaling also confers stress-avoidance advantages. This dual function makes BR pathway genes unusually efficient breeding targets, though BR-dependent stress regulation in cereals remains only partially mapped (Zolkiewicz and Gruszka, 2024).

SLs’ stress role runs substantially through ABA crosstalk given their shared carotenoid biosynthetic origin both pathways branch from the same precursor pool, creating an inherent coupling between SL and ABA production during drought (Haider et al., 2018). Exogenous application of the synthetic strigolactone GR24 to salt-stressed rice seedlings enhances antioxidant enzyme activity and restores disrupted chloroplast structure and gas-exchange parameters (Duan et al., 2025).

### 4.5 Emerging hormonal regulators

Beyond the nine canonical phytohormones, melatonin, whose classification as a confirmed classical plant hormone remains under active debate and is generally considered a hormone-like signaling molecule rather than a canonical phytohormone, has accumulated substantial evidence as a rice stress regulator. A comprehensive 2025 review frames it as a multifunctional signal operating through cross-regulatory networks with the canonical hormones (Chung et al., 2025). Exogenous melatonin applied to rice under simultaneous salt and drought stress measurably reduces oxidative damage and upregulates *OsSOS, OsNHX, OsHSF*, and *OsDREB* genes (Khan et al., 2024) a multi-pathway activation pattern consistent with melatonin functioning as a genuine upstream signal rather than a simple antioxidant.

**Table 1**, presented below, provides a systematic integration of phytohormone priming approaches across biotic and abiotic stress categories in rice, directly addressing the Special Issue focus on hormone priming as a strategy for crop resilience. For each priming study, the table documents the hormone applied, the stress condition addressed, the delivery method, the molecular mechanism activated, and the physiological or agronomic outcome achieved.

## 5 Phytohormone crosstalk under combined environmental stresses

### 5.1 ABA-JA interactions

The ABA-JA axis is the most studied hormone pairing in rice under abiotic stress, and its defining feature is conditional bidirectionality. The mechanistic spine runs through the JAZ-MYC2 module: under drought, ABA-activated SnRK2 kinases phosphorylate transcription factors that induce JA-biosynthesis genes, elevating JA-Ile and triggering COI1-dependent JAZ degradation, which frees *OsMYC2* and *OsMYC3* to activate osmotic and defense responses cooperatively (Ma et al., 2024). *OsJAZ9* overexpression elevates both endogenous ABA and JA, reduces stomatal density, and narrows leaf width to reduce transpirational water loss suggesting, based on a single study in wheat, that a JAZ protein can coordinately modulate both pathways (Mega et al., 2019). The E3 ubiquitin ligase *OsPUB16* simultaneously represses both ABA and JA biosynthesis through the SAPK9-*OsMADS23*-*OsAOC* module, with *OsPUB16* knockout producing stomata that close more efficiently under water deficit (Lv et al., 2022).

Transcriptome analysis of rice responding to combined ABA pretreatment and BPH infestation found that exogenous ABA upregulated JA-biosynthesis genes including *OsAOS2, OsOPR*, and *OsACOX*, while exogenous JA reciprocally elevated ABA synthesis providing the molecular basis for why drought-stressed rice sometimes shows enhanced rather than compromised BPH resistance (Li et al., 2022; Cao et al., 2025).

### 5.2 ABA-SA crosstalk

The ABA-SA relationship is predominantly antagonistic in the context of pathogen, though the degree of antagonism is concentration- and context-dependent defense. ABA promotes susceptibility to Xoo by suppressing SA-mediated defenses downstream of *OsWRKY13* but upstream of *OsNPR1*, and overexpression of *OsNPR1* fully blocks ABA-induced susceptibility placing the antagonism point precisely between these two regulators (Xu et al., 2013). The molecular mechanism involves ABA-induced proteasome-mediated degradation of NPR1 itself: ABA treatment reduces NPR1 protein levels while ABA-deficient mutants accumulate significantly more NPR1 than wild-type plants (Tian et al., 2025). The growth dimension adds a separate layer: SA supplied simultaneously with ABA completely blocks ABA’s inhibition of shoot growth and cell cycle progression by preventing ABA-induced expression of CDK inhibitors *OsKRP4, OsKRP5*, and *OsKRP6* (Meguro and Sato, 2014).

### 5.3 JA-SA antagonism and synergy

JA and SA are conventionally described as antagonists, canonical biotrophic-defense (SA) versus necrotrophic/herbivore-defense (JA). The antagonism runs through NPR1: cytosolic NPR1 activity mediates SA’s suppression of JA-dependent defenses, while JA activates ANAC019, ANAC055, and ANAC072, which bind directly to the ICS1 promoter and suppress SA biosynthesis (Pandey et al., 2025). In rice, *OsEIL2* loss-of-function mutants show enhanced resistance to both Xoo and M. oryzae but enhanced susceptibility to R. solani, suggesting OsEIL2 normally maintains the SA-JA balance point (Zhao et al., 2024).

Crucially, the antagonism is concentration- and context-dependent rather than absolute. In antiviral immunity, *OsNPR1* activates JA signaling by disrupting the *OsJAZ-OsMYC2* complex, driving cooperative immunity (Zhang et al., 2023b). Multiple unrelated viral proteins from rice stripe virus, southern rice black-streaked dwarf virus, and rice stripe mosaic virus all convergently target this *OsNPR1*-mediated SA-JA synergism as their virulence strategy. phylogenetically compelling argument that this synergistic relationship is not incidental but represents a genuine immune output that pathogens have specifically evolved to disrupt.

### 5.4 Ethylene-dependent networks

Ethylene occupies a structural position in hormone crosstalk that is qualitatively different from the other hormones: rather than being primarily a stress hormone in its own right, it functions as a context-setter determining how other hormone systems are interpreted (Zhang et al., 2026). Its signal transduction cascade, membrane receptor to *OsCTR2* to *OsEIN2* to *OsEIL1/OsEIN3* to AP2/ERF transcription factors produces the diverse outputs that include aerenchyma formation, internode elongation, and multi-hormone coordination through the large ERF family (Qin et al., 2024). *OsERF92* negatively regulates tolerance to both *M. oryzae* and salt stress, while overexpression of the barley ERF HvRAF in rice simultaneously enhances drought tolerance, salinity tolerance, and bacterial blight resistance demonstrating that a single ERF-type transcription factor can produce genuine multi-stress resilience (Hwang et al., 2026).

### 5.5 Growth-defense trade-offs

The growth-defense trade-off is the most consequential practical manifestation of hormone crosstalk in rice breeding. It arises structurally from overlapping use of DELLA and JAZ as shared co-repressors across the GA (growth) and JA (defense) pathways: the system is structurally zero-sum maximal activation of both pathways simultaneously through the same DELLA-JAZ interface is not achievable (Kazan and Manners, 2012). In rice specifically, BPH feeding reduces endogenous bioactive GA by activating *OsGA2ox3* and *OsGA2ox7* through JA-activated MYC2, producing the growth penalty that accompanies JA-mediated BPH resistance (Frungillo, 2023). That this penalty can be dissociated from the resistance itself is the central finding of the *OsJAZ10* frameshift study (Li et al., 2024a). Three distinct breeding approaches are now recognized for addressing these trade-offs: manipulation of susceptibility genes, manipulation of R genes with broad-spectrum pleiotropic effects, and direct hormonal manipulation (Gao et al., 2024; Xie et al., 2024a).

### 5.6 Hormonal network reprogramming during multi-stress exposure

When more than two stresses occur simultaneously, the pairwise models in Sections 5.1-5.5 become insufficient. Multi-stress exposure does not simply add pairwise interactions; it reprograms the hormonal network as a system, producing transcriptional outcomes that cannot be predicted from single-stress or dual-stress responses alone (Suzuki et al., 2014). *WRKY* transcription factors are the most extensively documented multi-stress hub. *WRKY13* functions as a bidirectional molecular switch simultaneously inhibiting drought-associated *SNAC1* and regulating SA-pathway immunity through differential promoter targeting (Ma et al., 2024). *OsWRKY76* positively regulates drought tolerance through *OsbHLH148*-mediated JA signaling (Zhang et al., 2023b). *OsMYB30* directly activates *OsPAL6* and *OsPAL8*, promoting both lignin and salicylic acid accumulation and enhancing resistance to BPH while contributing to cell wall reinforcement (Imran et al., 2025).

The integrated picture is consistent: under combined stress, rice converges multiple stress inputs onto a small number of shared transcription factors principally *WRKY, MYB*, MYC2/bHLH, NAC, and *bZIP* family members that operate as hormonal network arbitrators rather than linear signal transducers (Ganie et al., 2024; Aswathi et al., 2025). The cross-omics data in **Table 2** (Section 6.7) reinforce this: the (Vemanna et al., 2019) meta-analysis identified shared promoter motifs for ABA/JA/SA responses as the convergence point; the (Kang et al., 2025) framework introduced a quantitative ‘Total Hormonal Sensitivity Index’ to rank hormone-pathway responsiveness; and the (Yang et al., 2026) hormonomics study found that hormone balance, not individual magnitudes, predicts combined-stress seedling emergence. This convergence-onto-shared-hubs model has a direct engineering implication: targeting hub transcription factors rather than individual hormone pathway genes may be the more efficient route to combined-stress tolerance, while carrying higher pleiotropy risk. The tension between these two outcomes is the central engineering challenge addressed in Section 8.

**Table 2:** Representative multi-omics studies investigating hormone-mediated stress responses in rice (*Oryza sativa* L.).

#	Omics Platform(s)	Stress/Treatment	Genotype(s)	Key Hormone-Related Finding	Reference
1	Transcriptomics (meta-analysis)	Drought + bacterial blight (Xoo)	Multiple genotypes	Shared ‘core’ stress response activates ABA/JA/SA genes regardless of stress identity, forcing both stresses to compete for the same hormonal signaling capacity	(Vemanna et al., 2019)
2	Targeted hormonomics (LC-MS/MS, 15 hormones) + transcript validation	Combined drought + heat (reproductive stage)	AVT2–5315 vs. AC35027	Auxin/JA balance (not magnitude) tracked with spikelet fertility; ethylene/MeJA dominance tracked with sterility	(Da Costa et al., 2021)
3	Comparative proteomics (2-DE)	ABA pretreatment (priming) + salt	Rice seedlings	40 protein spots uniquely induced only in ABA-primed plants direct proteomic signature of the primed state	(Li et al., 2010)
4	WGBS methylome + transcriptome + small RNA	Drought, salinity (contrasting genotypes)	IR64, Nagina22, Pokkali	DNA methylation differences over hormone-responsive loci track tolerance class; epigenomic–transcriptomic integration	(Garg et al., 2015)
5	RNA-seq + WGBS + lncRNA + HPLC hormone quantification	Repeated drought/re-watering (priming cycles)	cv. Zhonghua 11	ABA/JA dynamics and DNA methylation jointly regulate stress-memory transcripts across priming cycles	(Li et al., 2019)
6	Comparative genomics + transcriptomics + widely-targeted metabolomics	Combined low-temperature + anaerobic stress (germination)	Dongxiang wild rice (O. rufipogon)	10,593 DEGs identified; core *DREB* genes coordinate energy/antioxidant pathways; 889 differentially accumulated metabolites	(Wang et al., 2025a)
7	Transcriptomics + metabolomics	Salinity + exogenous choline chloride priming	Two rice cultivars	Choline-chloride priming activates distinct transcriptional cascades alongside phytohormone signaling and antioxidant pathways	(Huo et al., 2024)
8	Untargeted metabolomics (WGCNA) + transcriptomics + targeted hormonomics (LC-MS/MS)	Hypoxic deep-sowing stress (germination/emergence)	CS2022 vs. HXR450	High-vigour line maintains 2x higher GA20/ABA ratio; JA ‘signal-buffering’ mechanism dissociates JA-biosynthesis transcripts from bioactive JA-Ile levels	(Yang et al., 2026)
9	Transcriptomics + targeted hormonomics + mathematical modeling	Thiocyanate (industrial pollutant) stress	cv. XZX 45	Tissue-specific hormonal homeostasis disruption quantified via novel ‘Total Hormonal Sensitivity Index’ framework	(Kang et al., 2025)

WGBS, whole-genome bisulfite sequencing; HPLC, high-performance liquid chromatography; WGCNA, weighted gene co-expression network analysis; LC-MS/MS, liquid chromatography-tandem mass spectrometry; 2-DE, two-dimensional gel electrophoresis; DEGs, differentially expressed genes; snRNA-seq, single-nucleus RNA sequencing; stRNA-seq, spatial-temporal RNA sequencing; VIP(m), Modified Variable Importance in Projection.

## 6 Multi-omics approaches revealing hormone priming networks

### 6.1 Transcriptomics

RNA-seq has become the default entry point for stress-hormone studies in rice. A 2024 integrated review of transcriptomic and proteomic stress studies documented how RNA-seq has mapped drought, salinity, and heat stress responses including the finding that drought stress specifically reprograms GA signaling and ABA catabolism in florets in a developmental-stage-dependent manner (Aslam et al., 2024). Alternative splicing represents a major underappreciated layer: a systematic RNA-seq data-mining study of rice leaves under heat stress identified 17,143 heat-responsive differentially expressed genes alongside 2,162 genes showing differential alternative splicing events, many encoding key transcriptional regulators demonstrating that the stress transcriptome’s diversity substantially exceeds what expression-level analyses alone reveal (Vitoriano and Calixto, 2021). Integration of ATAC-seq with RNA-seq in heat-stressed rice leaf tissue identified three key transcription factor genes *OsbZIP14*, *OsMYB2*, and *OsHSF7* with both chromatin accessibility and expression altered under heat (Qiu et al., 2023). In the priming context, transcriptomics resolves distinct priming-specific transcriptional programs: oligosaccharide priming of salt-stressed rice generates 4,125 DEGs under salinity enriching photosynthesis, cell wall synthesis, and signal transduction pathways not enriched under stress alone (Xu et al., 2025).

### 6.2 Proteomics

The most critical advance enabling modern rice stress proteomics is the shift from 2-dimensional electrophoresis to LC-MS/MS-based mass spectrometry approaches that can detect post-translational modifications, particularly phosphorylation the primary mechanism through which ABA, MAPK, and SnRK2 signal transduction actually operates. A 2024 phosphoproteomics meta-analysis identified 15,565 phosphosites on serine, threonine, and tyrosine residues across rice proteins, linking phosphorylation motifs to specific biological processes and kinase families providing the first pan-rice map of phosphorylation-based signaling variation (Ramsbottom et al., 2024). An earlier phosphoproteomics study mapped ABA-induced phosphorylation in rice seedlings, identifying 2,271 phosphosites including SnRK2-pathway components, and discovering that ABA antagonistically regulates brassinosteroid signaling via BR receptor inhibition, a hormone-crosstalk finding invisible to transcript-level analysis alone. In the biotic-stress interface, comprehensive phosphoproteomics of rice responding to Xoo infection identified 762 differentially phosphorylated proteins at 24 hours post-infection, mapping the kinase cascade that translates early immune recognition into transcriptional reprogramming (Hou et al., 2015).

### 6.3 Metabolomics

Phytohormones are themselves metabolites, and their absolute concentrations not just their pathway gene expression levels determine signaling outcomes. LC-MS/MS-based targeted hormonomics can now quantify 15 or more hormone species simultaneously in a single rice extract (Da Costa et al., 2021; Yang et al., 2026). Metabolite and phytohormone profiling of deepwater rice under partial submergence confirmed that GA-20 and GA-1 bioactive forms accumulate rapidly during escape-strategy elongation while ABA levels decline providing direct metabolomics confirmation that the GA-ABA balance shift drives the adaptive elongation response (Fukushima et al., 2020). Untargeted metabolomics using WGCNA integrated with targeted hormonomics resolved the GA/ABA/JA balance in high- versus low-vigor rice seedlings under hypoxic direct-seeding stress, showing that the 2× higher GA20/ABA ratio in high-vigor lines is a measurable metabolite-level hormonal state that persists across the critical germination window (Yang et al., 2026) (**Table 2**).

### 6.4 Epigenomics

Epigenomics captures the chromatin regulation layer that explains why primed plants respond differently to a second stress encounter. The rice epigenomics toolkit now includes WGBS for DNA methylation, ChIP-seq for histone modifications, ATAC-seq for chromatin accessibility, and small RNA profiling for RNA-directed DNA methylation (RdDM). Cold-tolerant rice variety P427 shows significantly higher numbers of genes with altered DNA methylation under low-temperature stress than other varieties, with increased expression correlating with reduced methylation at those loci directly linking epigenomic state to cold tolerance phenotype (Li et al., 2025). Histone modifications at ABA-responsive loci maintaining their H3K4me3 poised state are the molecular definition of the primed condition, and CRISPR-dCas9 epigenome editing targeting methylation marks at *OsDREB1* stress-responsive loci improves rice drought tolerance by 25% the first quantified tolerance improvement from targeted epigenome editing (Qadir et al., 2026).

### 6.5 Single-cell omics

Single-cell RNA sequencing (scRNA-seq) has resolved a major limitation of bulk transcriptomics: the assumption that all cells of a given tissue respond uniformly to stress. The first comprehensive single-cell transcriptomic landscape of rice mapped cell-type-specific proportional changes under abiotic stress, revealing that canonical stress-response genes are differentially regulated at the cell-type level rather than uniformly induced (Wang et al., 2021b). A landmark 2025 Nature study used combined scRNA-seq and spatial transcriptomics of rice roots grown in soil versus gel conditions to show that outer root cell types undergo major transcriptional changes related to cell wall integrity and defense in response to heterogeneous soil stress completely invisible in gel-condition experiments and bulk RNA-seq (Zhu et al., 2025). Most directly relevant to this review’s biotic-stress theme, a 2025 Advanced Science study combining snRNA-seq with spatial-temporal transcriptomics (stRNA-seq) of *M. oryzae*-infected rice leaves found that approximately 75% of differentially expressed genes detected at single-cell resolution were not detectable by bulk RNA-seq (Wang et al., 2025b) It should be noted that single-cell transcriptomics carries inherent technical limitations, including potential transcriptional changes introduced during cell isolation (dissociation artifacts) and cell-size-based recovery biases. Notwithstanding these caveats, the study demonstrated that immune-response gene expression during blast infection is dominated by ‘lower-profile’ DEGs that bulk approaches systematically miss.

### 6.6 Spatial transcriptomics

**Figure 2:**
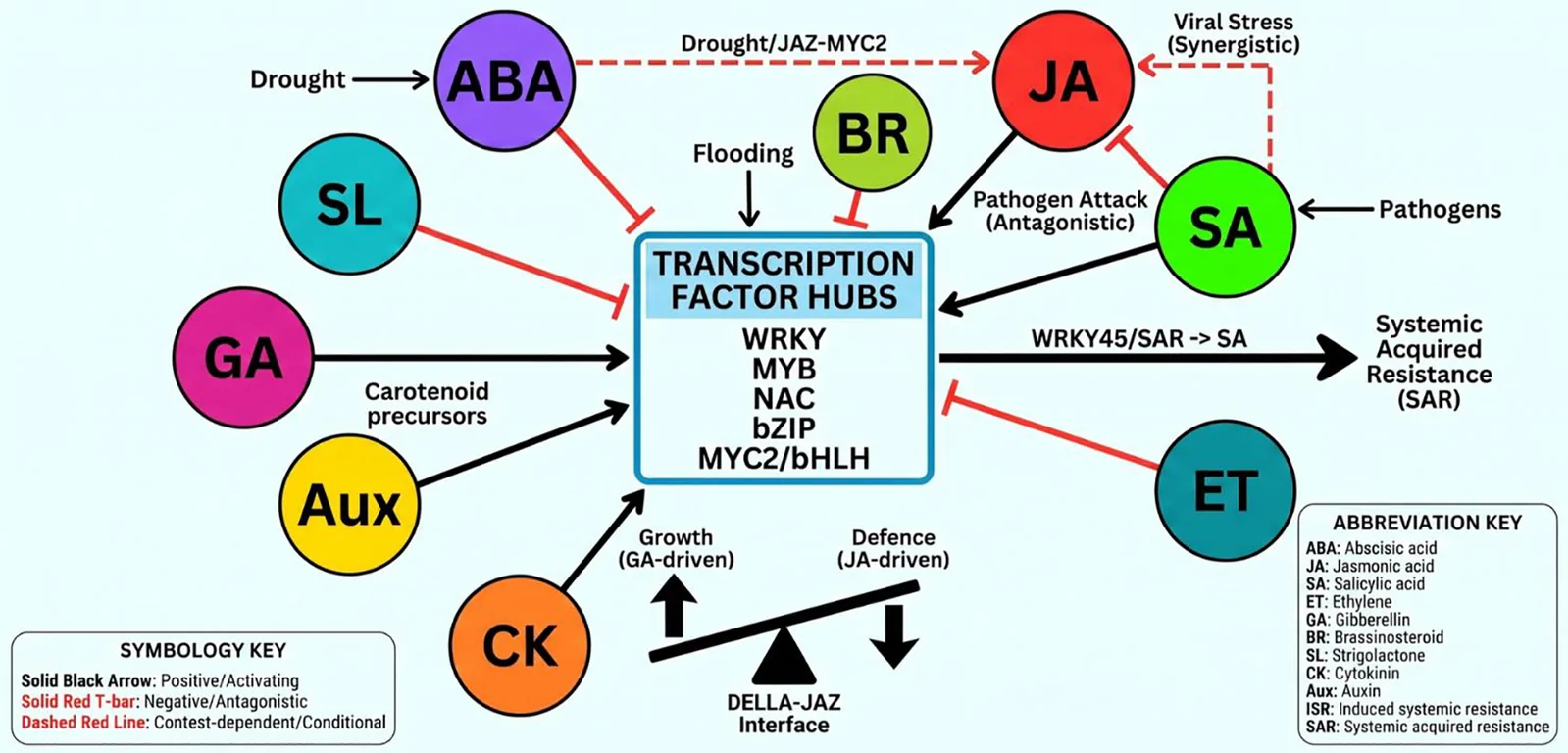
Phytohormone crosstalk network in rice under combined biotic and abiotic stress. This network maps the interactions between nine major hormone classes (ABA, JA, SA, ET, GA, BR, SL, CK, and Aux) during multi-stress adaptation. Arrows indicate positive (activating) interactions; blunted lines represent negative (antagonistic) interactions; and dashed lines denote context-dependent (conditional) relationships. Key integration points include: • Signaling Hubs: Transcription factor families (WRKY, MYB, MYC2/bHLH, NAC, *bZIP*) act as central network arbitrators to integrate multiple hormone inputs. • Trade-off Node: The DELLA-JAZ shared co-repressor interface mediates the zero-sum competition between GA-dependent growth and JA-dependent defense. • Documented Synergies: Includes ABA-JA (under drought via JAZ-MYC2), SL-ABA (via shared carotenoid precursors), and SA-CK (in *WRKY45*-mediated SAR priming). • Documented Antagonisms: Includes ABA-SA (via NPR1 degradation), GA-ABA and ET-ABA (in growth/flooding coordination), and the context-dependent JA-SA balance. ABA, abscisic acid; JA, jasmonic acid; SA, salicylic acid; ET, ethylene; GA, gibberellin; BR, brassinosteroid; SL, strigolactone; CK, cytokinin; Aux, auxin; ISR, induced systemic resistance; SAR, systemic acquired resistance.

Spatial transcriptomics preserves tissue architecture information, revealing not just which genes are expressed in which cell types but where in the tissue those cells are located. This is particularly important for hormone signaling research because many phytohormone signals including auxin, ABA derived from phloem, and SA moving through vascular tissue are spatially graded rather than uniformly distributed. A comparative spatial transcriptomics study of rice roots under drought identified *HMGB1* as a key regulator of root adaptation and documented that ABA originating from phloem tissues integrates local mechanical stress perception with whole-root adaptive responses through systemic hormone signaling (Zhong et al., 2025). The (Wang et al., 2025b) study further confirmed that SA and JA signaling pathway genes during blast infection showed similar expression patterns across all cell types indicating hormone signaling in rice immunity operates as a systemic, tissue-uniform transcriptional response rather than a cell-type-specific precision signal (**Figure 2**).

### 6.7 Integrated multi-omics analysis

No individual omics platform generates a complete picture of hormone priming networks. The studies assembled in **Table 2** span six omics platforms across nine stress combinations from 2010 to 2026, making three convergent points: (1) hormonomics data consistently show that hormone balance the ratio between competing signals, especially GA20/ABA and JA-Ile/bioactive JA predicts stress outcomes more reliably than the absolute concentration of any single hormone; (2) epigenomics layers show that tolerance-class-specific methylation differences at hormone-responsive loci are detectable at the whole-genome level and persist across stress cycles; and (3) the integrated multi-omics studies generate hypotheses that no single layer could produce, including the (Kang et al., 2025) mathematical modeling approach that introduces predictive, quantitative hormonal sensitivity models usable to rank engineering targets before experimental validation. A comprehensive 2025 review of multi-omics integration for rice disease resistance frames the current frontier: AI and network tools including WGCNA, graph-based network construction, and machine learning are increasingly enabling identification of hormone-pathway hub genes that no individual experiment could resolve. It should be noted that multi-omics integration carries inherent limitations: data heterogeneity across studies with differing cultivars and growth conditions limits cross-study comparisons; AI models trained in controlled environments may underperform in field conditions; and the biological interpretability of black-box machine learning models remains a challenge. Standardized protocols and open-access data deposition remain important needs (Rajamuthu et al., 2025).

### 6.8 Multi-omics guided discovery of engineering targets

The most valuable engineering targets are those identified as nodes of hormone-network convergence across multiple omics layers simultaneously. *OsNCED3* exemplifies this principle: transcriptomics identifies it as a consistently drought-upregulated gene; hormonomics confirms its rate-limiting role in ABA biosynthesis; phosphoproteomics identifies its regulation through ABA-dependent kinase cascades; and mathematical modeling (Kang et al., 2025) ranks the ABA biosynthesis pathway as the highest-sensitivity hormone pathway under combined stress. Concordance across four independent data streams provides a substantially stronger evidence basis for engineering target prioritization than any single experimental observation could provide.

Weighted gene co-expression network analysis (WGCNA) applied to rice transcriptomics datasets identifies hub genes whose expression co-varies with hormone-balance phenotypes across multiple stress combinations, and these hub genes are systematically more likely to produce multi-stress tolerance gains when edited than randomly selected stress-responsive genes (Rajamuthu et al., 2025). Application of graph neural networks and machine learning classifiers to integrated multi-omics data is now enabling prediction of which gene edits will produce tolerance improvements versus which will produce pleiotropic developmental penalties, shifting genetic engineering from hypothesis-driven to data-driven target selection.

The practical multi-omics guided target discovery workflow is: (1) integrate transcriptome, phosphoproteome, metabolome, and methylome data across diverse stress conditions; (2) apply network analysis to identify hormone-pathway hub genes showing concordant multi-layer evidence; (3) apply mathematical modeling to quantify the hormonal sensitivity of identified targets; (4) validate top-ranked targets through multiplex CRISPR editing in controlled conditions; (5) advance validated targets to field trials. This proposed framework is technically feasible with currently available tools and datasets, and represents the most promising near-term pathway to rationally designed combined-stress tolerance in rice, and is the operational framework through which Section 7 metabolic engineering strategies were prioritized in this review.

## 7 Metabolic engineering strategies for enhancing stress tolerance

### 7.1 Engineering hormone biosynthesis pathways

**Figure 3:**
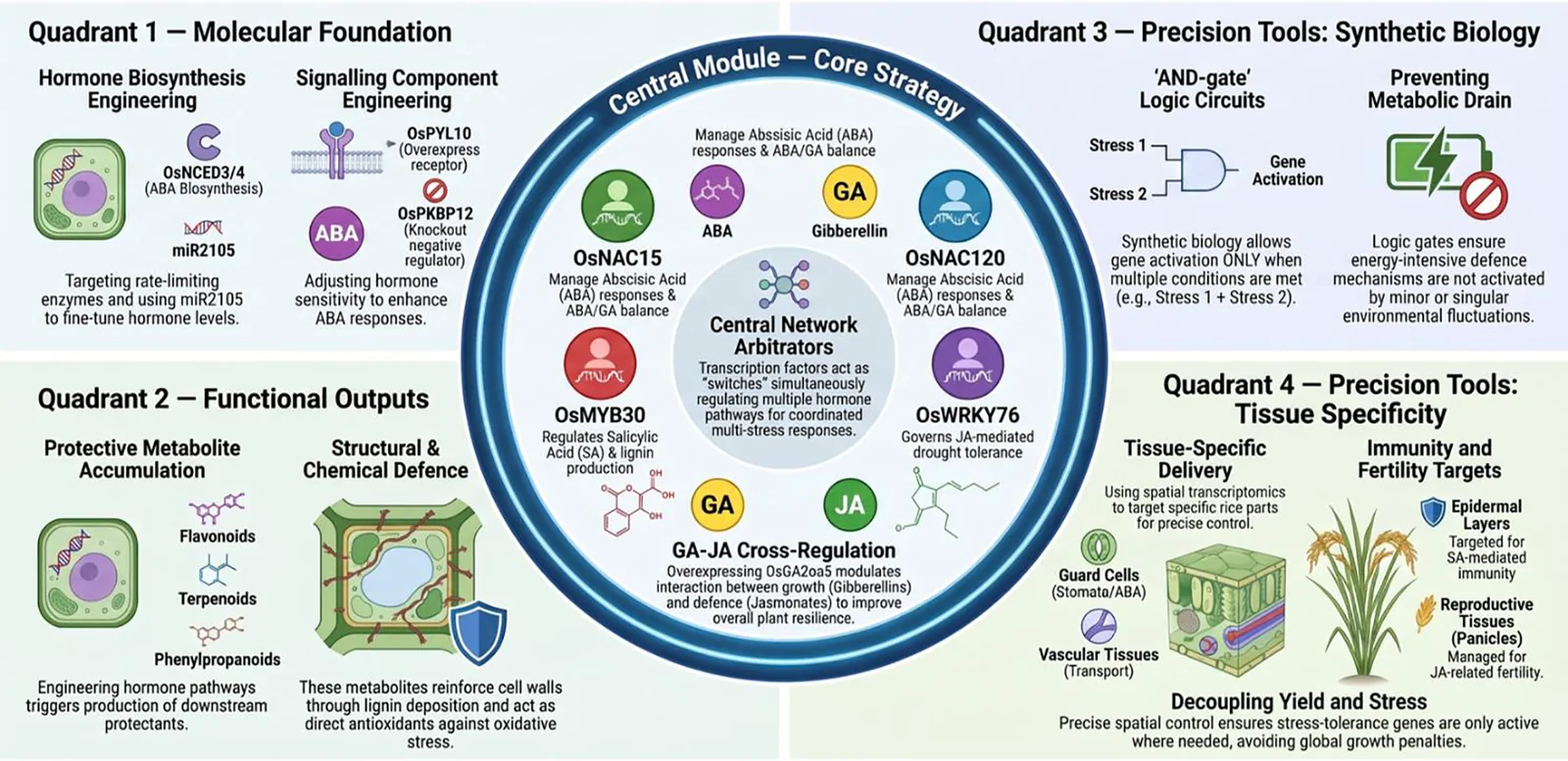
Integrated metabolic engineering strategies for hormone-mediated stress tolerance. This schematic outlines a four-pillared approach to crop resilience centered on a regulatory core. • Core Strategy: Central network arbitrators (*OsNAC15, OsNAC120, OsMYB30*, and *OsWRKY76*) act as molecular switches to coordinate multi-stress responses across various hormone pathways. • Molecular Foundation: Targets hormone biosynthesis (e.g., *OsNCED3/4*) and signaling sensitivity (e.g., *OsPYL10*) to fine-tune environmental responses. • Functional Outputs: Focuses on accumulating protective secondary metabolites (flavonoids, terpenoids) that provide structural and chemical defense. • Precision Tools: Utilizes Synthetic Biology (AND-gate logic circuits for conditional gene activation) and Tissue-Specific Delivery (targeting guard cells or vascular tissues) to decouple stress tolerance from growth penalties.

The most direct metabolic engineering approach targets rate-limiting enzymatic steps in hormone biosynthesis pathways to modulate endogenous hormone levels without introducing heterologous genetic regulatory elements. In the ABA pathway, *OsNCED3* encoding the key rate-limiting enzyme 9-cis-epoxycarotenoid dioxygenase is constitutively expressed in rice tissues and highly induced by NaCl, PEG, and H_2_O_2_ stress. Overexpression of *OsNCED3* in transgenic plants enhances water stress tolerance and increases endogenous ABA content, while nced3 mutants show increased sensitivity to water stress and increased stomatal aperture under water stress (Huang et al., 2018). Fine-tuning ABA levels through miRNA-mediated regulation also shows promise: *miR2105* regulates ABA biosynthesis via the *OsbZIP86-OsNCED3* module, contributing to drought tolerance. The *miR2105* pathway illustrates that biosynthesis engineering does not require direct gene overexpression targeting regulatory microRNAs that control biosynthesis genes achieves similar outcomes with potentially fewer pleiotropic effects (**Figure 3**).

In the GA pathway, *OsGA2ox8* overexpression activates JA signal transduction by inhibiting the expression of JAZ proteins, producing a GA-JA cross-pathway effect that simultaneously modulates secondary metabolism through anthocyanin and flavonoid biosynthetic genes (Wang et al., 2021a). The practical lesson is that hormone biosynthesis engineering rarely has single-pathway effects: altering ABA biosynthesis affects GA homeostasis, and vice versa, because the two pathways share carotenoid precursor pools. This metabolic interconnection means biosynthesis-level engineering of one hormone often inadvertently reprograms others a complexity that tissue-specific promoters (Section 7.6) and synthetic gene circuits (Section 7.5) are specifically designed to manage.

A critical priority for future metabolic engineering efforts is specifically targeting combined biotic-abiotic stress scenarios, which remain underrepresented in the current literature. For example, engineering drought tolerance via *OsNCED3* overexpression must be evaluated simultaneously against Magnaporthe oryzae susceptibility, since elevated ABA can compromise SA-mediated blast resistance. Similarly, engineering JA-pathway components for BPH resistance (e.g., *OsJAZ10* frameshift) should be assessed under concurrent heat stress, which is emerging as a co-occurring pressure during the reproductive stage. The development of multiplex editing strategies specifically optimizeoptimized for combined-stress scenarios, rather than single-stress performance, should be the central objective of the next generation of hormone-pathway engineering programs.

### 7.2 Engineering hormone signaling components

Rather than altering hormone levels, engineering signaling components allows engineering of hormone sensitivity the degree to which a given hormone concentration translates into a downstream response. The clearest current example is engineering of ABA receptors: overexpression of the ABA receptor *OsPYL10* under the stress-inducible RD29A promoter in indica mega rice variety MTU1010 produces transgenic lines with 2-3.3-fold higher ABA accumulation in flag leaf at anthesis under non-stress conditions and significantly higher survival rates under cold stress at the seedling stage (Kumar et al., 2020). The receptor overexpression approach hypersensitizes the ABA pathway without requiring elevated ABA production, achieving tolerance at a lower metabolic cost than biosynthesis-pathway engineering. Engineering of signaling kinases provides complementary gains: *SAPK2* (a SnRK2-type kinase downstream of ABA receptor activation) overexpression enhances drought tolerance, while *OsFKBP12* a negative regulator of ABA signaling knockout lines show enhanced ABA sensitivity and drought tolerance, upregulating *OsNCED3, OsSNAC1*, and *OsDREB2A* (Jiang et al., 2025).

### 7.3 Manipulation of transcription factors

Transcription factors that operate at multiple hormone-pathway intersections simultaneously represent the most efficient metabolic engineering targets for combined-stress tolerance, since a single edit can shift multiple hormone outputs in a coordinated way. *OsNAC15* activates ABA biosynthesis, with studies reporting enhanced tolerance to salt and drought stress in transgenic lines, while *OsNAC120* has been shown to improve drought resistance by modulating both ABA and GA; however, it should be noted that phenotypic outcomes for both genes may vary substantially depending on genetic background, growth conditions, and the specific cultivar used (Ao et al., 2024; Xie et al., 2024b). These NAC-family examples illustrate the hormone-network arbitrator function described in Section 5.6: they do not exclusively regulate one pathway but instead coordinate the balance between competing hormone signals. *OsMYB30* simultaneously activates *OsPAL6* and *OsPAL8*, promoting both lignin and salicylic acid accumulation and enhancing BPH resistance while contributing to cell wall reinforcement a single MYB transcription factor connecting JA/SA biotic defense with structural abiotic-stress adaptation (Imran et al., 2025). The risk of transcription factor engineering is the converse of its efficiency: because these factors coordinate multiple pathways, edits are more likely to produce unintended pleiotropic developmental or metabolic consequences than edits to single-hormone biosynthesis genes.

### 7.4 Engineering secondary metabolism

Secondary metabolites produced downstream of JA, SA, and brassinosteroid signaling including flavonoids, phenylpropanoids, terpenoids, and alkaloids serve dual functions as direct stress protectants and as volatile or exuded signals that coordinate systemic defense. Engineering secondary metabolism through hormone pathways therefore simultaneously improves both primary stress defense and the systemic priming signals that protect distal tissues. Overexpression of *OsGA2ox8* activates JA signal transduction and simultaneously modulates flavonoid and anthocyanin biosynthesis, demonstrating a direct hormone-to-secondary-metabolism engineering connection in rice (Wang et al., 2021a). Plant metabolic engineering for enhanced nutrition and food security increasingly focuses on dual-purpose crops in which secondary metabolite enhancement and stress tolerance co-occur: overexpressing the Orange gene variant IbOr-R96H boosts carotenoid content and antioxidant activity while increasing ABA levels in sweet potato, and the rice *OsOR* ortholog achieves similar dual benefits in rice, making it one of the few examples of a truly dual-function metabolic engineering target (Kim et al., 2021; Kaya, 2025).

### 7.5 Synthetic biology approaches

Synthetic biology moves beyond modification of endogenous pathways to construction of entirely novel gene circuits that program precise, conditional hormone responses impossible to achieve with natural regulatory elements. Unlike constitutively expressed transgenes, synthetic gene circuits can integrate multiple input signals to achieve sophisticated transcriptional logic activating a hormone biosynthesis gene only when both a specific stress is detected and a specific developmental stage has been reached, for example (Lloyd et al., 2025). In the Arabidopsis context, synthetic promoters driving ABA receptor genes (CARK1 and RCAR11) have been designed to respond to both ABA and *DREB2A* inputs through AND-gate logic, producing drought tolerance only under conditions where both signals are simultaneously present and thus avoiding constitutive activation costs (Ge et al., 2018). A 2025 review of synthetic gene circuits in plants identifies the C4 rice project as the field’s most ambitious application using synthetic promoters for multiplex cell-type specificity of target genes and notes that conditional control of expression through logic gate circuits could substantially reduce metabolic burden (Lloyd et al., 2025). For hormone-priming applications specifically, the most tractable near-term target is stress-inducible delivery of JA or ABA biosynthesis genes under characterized stress-responsive promoters, which avoids the constitutive activation that historically produced yield penalties in early phytohormone engineering efforts.

### 7.6 Tissue-specific metabolic engineering

The tissue specificity of hormone signaling with ABA acting primarily in stomatal guard cells, JA in phloem-associated tissues, and SA in the epidermis and vascular bundle means that constitutive hormone pathway engineering routinely produces off-target phenotypes in tissues where the engineered hormone level is not beneficial. Tissue-specific promoters tailored to guard cells, leaf epidermis, roots, or reproductive organs represent the most direct solution. Rice tissue-specific promoters that are both well-characterized and condition-dependent allowing activation only in the target tissue and only under stress represent the highest-value targets for this approach. Spatial transcriptomics data (Section 6.6) are now providing the tissue-specific expression maps needed to identify appropriate promoter candidates for each hormone pathway: ABA hormone transducers and transporters concentrated in ovular vascular trace cells (Zhong et al., 2025) suggest vascular-specific promoters as delivery vehicles for ABA-pathway engineering targeting grain fill under drought (**Figure 3**).

## 8 Genome editing and precision breeding for hormone-mediated stress tolerance

### 8.1 CRISPR-Cas systems

CRISPR-Cas9 has become the dominant precision tool for hormone-pathway engineering in rice, offering transgene-free editing capability, multiplexing potential, and a rapidly expanding toolkit of Cas variants with improved specificity. CRISPR applications in rice have produced measurable gains across all major hormone pathways, with the most compelling examples combining enhanced stress tolerance with no yield penalty or even improved yield a combination historically impossible in conventional breeding for stress tolerance (Ahmed et al., 2021; Kaniganti et al., 2026; Malik et al., 2026). The gene targets represented in **Table 3** (*OsPYL9, OsDST, OsOLP1* for ABA pathway; *OsS5H1/2/3* for SA pathway; *OsJAZ10, OsJAZ12/13, OsCOI1b* for JA pathway) collectively demonstrate that hormone-pathway genes can be edited with high precision to produce specific phenotypic gains without disrupting the broader hormonal architecture when the edit is targeted appropriately.

**Table 3:** CRISPR/Cas9-edited rice genes associated with hormone-mediated stress tolerance.

#	Gene(s) edited	Hormone pathway	Editing outcome	Stress/Phenotype	Reference
1	*OsPYL9* (ABA receptor)	ABA	Knockout → elevated endogenous ABA, antioxidant activity, cuticular wax	Drought tolerance AND increased grain yield under both drought and well-watered conditions no yield penalty	(Usman et al., 2020)
2	*OsDST* (drought & salt tolerance)	ABA/stomatal regulation	366-bp deletion → wider leaves, lower stomatal density	Combined drought AND salinity tolerance via improved water retention	(Kumar et al., 2020)
3	*OsOLP1* (osmotin-like protein)	ABA biosynthesis	Knockout → reduced ABA, lignin, proline; overexpression → enhanced tolerance	Drought tolerance via ABA-mediated stomatal closure and lignin deposition	(Yan et al., 2023)
4	OsS5H1/2/3 (SA 5-hydroxylase, triple mutant)	Salicylic acid	Simultaneous triple knockout → elevated SA (blocks SA to 2,5-DHBA conversion)	Broad-spectrum resistance to bacterial blight AND rice blast simultaneously	(Liu et al., 2023)
5	*OsJAZ10* (frameshift to novel protein FJ10)	Jasmonate	CRISPR frameshift generates novel protein; non-canonical JA signaling activation	Enhanced brown planthopper resistance WITH NO growth penalty directly resolves growth-defense trade-off	(Li et al., 2024a)
6	*OsJAZ12*, *OsJAZ13*, *OsCOI1b*, *OsNINJA*	Jasmonate-ABA crosstalk	Four independent knockouts	Mutants reveal JA pathway genes regulate ABA metabolism via *OsNCED1/OsABA8ox1* direct genetic evidence of JA-ABA mechanistic coupling	(Mao et al., 2025)

### 8.2 Base editing

Base editing extends CRISPR precision beyond indels to programmable single-nucleotide transitions (C-to-T via cytosine base editors, or A-to-G via adenine base editors) without introducing double-strand DNA breaks. This is particularly valuable for hormone-pathway genes where specific point mutations rather than complete knockouts alter receptor affinity, enzyme kinetics, or transcription factor binding specificity in a tuneable way. Cytosine base editing has been applied in rice to convert C-G to T-A base pairs, achieving herbicide-resistant plants via the C287T mutation in acetolactate synthase (Shimatani et al., 2017; Tanveer et al., 2025). For hormone-pathway applications, the ability to install specific amino acid substitutions in receptor proteins analogous to naturally occurring gain-of-function mutations in ABA receptors without creating large deletions that might disrupt protein structure offers a technically superior route to receptor engineering compared with full knockouts (Tanveer et al., 2025).

### 8.3 Prime editing

Prime editing integrates Cas9 nickase with a reverse transcriptase guided by a prime editing guide RNA (pegRNA), enabling all 12 types of point mutations, small insertions, and small deletions without double-strand breaks and without donor DNA templates. Critically for complex crop improvement, prime editing can make up to 89% of known pathogenic variants correctable, a capability that translates in rice to the ability to install beneficial alleles rather than simply knock out unfavorable ones. (Lou et al., 2025 used prime editing in rice under heat stress to achieve up to 25% higher yields in multi-location field trials with some lines rescuing over 40% of heat-induced yield losses without affecting grain quality establishing field-scale validation of prime editing for climate-stress tolerance in rice (Lou et al., 2025; Al-Saadi et al., 2026). Modular assembly-based multiplex prime editing systems have achieved co-editing efficiency of 46.1% in the T0 generation in rice, simultaneously editing TFIIAy5 to xa5 and xa23 to Xa23SW11 to produce broad-spectrum resistance against multiple Xoo strains (Gupta et al., 2024).

### 8.4 Multiplex genome editing

Given the multi-pathway nature of combined-stress tolerance and the convergence of hormone crosstalk onto shared hubs, single-gene edits rarely produce the simultaneous, coordinated pathway adjustments that combined-stress resilience requires. Multiplex editing simultaneously targeting multiple genes in a single transformation is therefore the logical extension of CRISPR for hormone-network engineering. The *OsS5H1/2/3* triple knockout (**Table 3**) is the current benchmark for multiplexed hormone-pathway editing in rice, achieving broad-spectrum disease resistance through simultaneous SA-pathway reprogramming at three enzymatic steps (Liu et al., 2023). Quadruple prime editing of four genes simultaneously has been demonstrated in rice (QPE system), establishing technical feasibility for the kind of multi-pathway hormone engineering that combined-stress tolerance demands (Gupta et al., 2024). The CRISPR/Cas genome editing review for cereal crops identifies editing of disease susceptibility genes particularly SWEET-family sugar transporters hijacked by Xoo as one of the most effective multiplex strategies because it blocks pathogen exploitation without activating constitutive defense pathways (Oliva et al., 2019; Kaniganti et al., 2026).

### 8.5 Editing hormone regulatory genes

**Table 3** presents the six most informative CRISPR-edited rice genes associated with hormone-mediated stress tolerance, selected to represent three hormone pathways (ABA, SA, JA), diverse editing outcomes (knockout, frameshift, triple multiplex), and a range of targeted phenotypes from drought tolerance through broad-spectrum disease resistance to insect resistance (**Table 3**). The most important conceptual advance represented collectively by these six entries is that hormone-pathway editing can now be designed to decouple historically linked phenotypes: *OsPYL9* knockout decouples drought tolerance from yield penalty, *OsJAZ10* frameshift decouples BPH resistance from growth penalty, and the *OsJAZ12/13*/COI1b/NINJA quadruple knockout reveals the JA-ABA coupling mechanism that makes simultaneous drought and biotic stress tolerance feasible. Together, these examples validate the review’s central proposition that hormone-pathway engineering under combined-stress scenarios should target crosstalk nodes rather than individual pathway endpoints.

### 8.6 Future CRISPR directions

The next generation of CRISPR applications for rice hormone-pathway engineering extends well beyond simple knockout or knock-in editing. Four complementary approaches are currently transitioning from proof-of-concept to agricultural application, and each addresses a distinct limitation of the conventional CRISPR editing strategies described in Sections 8.1-8.5.

CRISPRa (CRISPR activation) uses catalytically inactive dCas9 fused to transcriptional activation domains, delivered through signal amplification systems such as SAM, SunTag, or the more recently developed MoonTag, to upregulate endogenous hormone-responsive genes without altering their DNA sequence, achieving programmable transcriptional priming that mimics hormone priming at the chromatin level (McLaughlin et al., 2025). CRISPRa targeting of the SlPR-1 promoter in tomato demonstrated that dCas9-mediated transcriptional activation drives epigenetic reprogramming through increased H3K4me3 deposition, augmenting SA-mediated immune pathways and SAR activation upon pathogen challenge without impacting key agronomic characteristics (McLaughlin et al., 2025). The direct applicability to rice SA-pathway priming engineering is clear: CRISPRa targeting of *OsNPR1* or *WRKY45* promoter regions could achieve the equivalent of SA priming as a stable, heritable genetic trait rather than a recurring agronomic input.

CRISPRi (CRISPR interference) uses dCas9 fused to transcriptional repressors or dCas9 epi-editors targeting H3K9me3 deposition, CpG methylation, and histone deacetylation to silence hormone-pathway negative regulators in a dose-tuneable and reversible manner. Silencing of JAZ repressors through CRISPRi allows reversible, dose-dependent JA pathway activation without the permanent indel that full CRISPR knockouts introduce, potentially enabling inducible JA priming responses that are activated by stress detection and then reset upon stress relief, avoiding the constitutive growth penalties that have historically accompanied JA-pathway engineering (McLaughlin et al., 2025).

Tissue-specific CRISPR delivery, guided by the spatial transcriptomics data described in Section 6.6, enables hormone-pathway edits to be restricted to the cell types where the edit is beneficial: vascular cells for ABA-pathway modifications targeting phloem-derived ABA signaling, guard cells for stomatal ABA regulation, epidermal cells for SA-pathway immunity priming, and reproductive organs for JA-mediated pollen fertility protection. CRISPR nanoparticle delivery systems capable of achieving tissue-specific transformation without Agrobacterium are now sufficiently advanced in rice to make this approach practically feasible for the near term (McLaughlin et al., 2025).

Multiplex prime editing targeting up to four hormone-regulatory genes simultaneously (QPE system, (Gupta et al., 2024 represents the most technically advanced current approach for engineering the hormone-balance phenotypes described throughout this review. The near-term objective is a multiplex prime editing strategy that simultaneously installs: *OsPYL9* loss-of-function (ABA pathway, drought tolerance without yield penalty), *OsS5H1/2/3* loss-of-function (SA pathway, broad-spectrum disease resistance), and *OsJAZ10* frameshift (JA pathway, insect resistance without growth penalty) in a single transformation event. This three-pathway simultaneous optimization would represent the first rice variety engineered specifically for the combined hormone-network balance that combined-stress tolerance demands.

## 9 Microbiome-assisted hormone priming

### 9.1 Plant growth-promoting rhizobacteria

Plant growth-promoting rhizobacteria (PGPR) have emerged as one of the most tractable field-scale delivery systems for hormone priming, circumventing both the regulatory challenges of genetic modification and the precision-dosing problems of exogenous chemical priming (Alshegaihi et al., 2026). PGPR produce or modulate all of the major phytohormones discussed in this review, including indole-3-acetic acid (IAA produced by approximately 80% of soil microorganisms including Pseudomonas sp., Bacillus sp., Burkholderia sp., and Rhizobium sp.), gibberellins, cytokinins, and ABA (Verma et al., 2024). PGPR-rhizobacteria additionally downregulate the effects of abiotic stresses by modifying the expression of genes associated with the biosynthesis of ethylene (ACO and ACS genes), jasmonate (MYC2), and SA (PR1), as well as genes encoding antioxidant enzymes and stress-associated transcription factors including NAC1 (Verma et al., 2024).

In rice-specific applications, Bacillus megaterium strains CACC109 and CACC119 isolated from ginseng fields modulate antioxidant-related and drought-responsive gene expression to protect rice from drought, exhibiting plant growth-promoting activities including phosphate solubilization, nitrogen fixation, IAA production, siderophore secretion, ACC deaminase activity, and exopolysaccharide production (Lee et al., 2024). Harnessing Bacillus subtilis and B. aryabhattai to combat salt stress in rice through phytohormone regulation has been demonstrated to regulate antioxidant defense, ion homeostasis, and photosynthetic parameters, with PGPR inoculation producing measurable improvements in AsA-GSH pathway activity and auxin-mediated growth enhancement under stress conditions (Alwutayd et al., 2024; Siddika et al., 2024).

### 9.2 Endophytes

Endophytic bacteria and fungi colonize internal rice tissues without causing pathological symptoms and produce hormones, volatiles, and secondary metabolites that prime host defense from within the plant. Unlike rhizobacteria that operate at the root-soil interface, endophytes can influence hormone homeostasis in aerial tissues including leaves and reproductive organs making them particularly relevant for priming against combined abiotic stresses that manifest most severely above ground (heat stress on pollen, drought stress on panicle water potential). The precision of endophytic hormone delivery is inherently superior to exogenous spraying: because endophytes reside within the apoplast, phloem, or intercellular spaces of specific tissues, the hormone-modifying activity they provide is automatically tissue-localized without requiring synthetic promoter engineering (Kunal et al., 2023). Bacterial cyclodipeptides produced by certain endophytes act as direct priming agents with documented antibacterial, antiviral, and immunostimulatory activity once perceived by the plant, providing a mechanistic basis for the broad-spectrum priming observed in many endophyte inoculation experiments (Jatana et al., 2024).

### 9.3 Microbial regulation of hormone homeostasis

PGPR regulation of hormone homeostasis extends beyond simple phytohormone production to sophisticated signaling that involves competition with plant ACC (1-aminocyclopropane-1-carboxylate, the ethylene precursor) for substrate, production of volatile organic compounds that trigger systemic ISR (induced systemic resistance) through JA-dependent pathways, and secretion of exopolysaccharides that modify root architecture and thereby alter auxin distribution gradients (AL-Huqail et al., 2025). ACC deaminase activity which cleaves plant-produced ACC before it can be converted to ethylene is particularly relevant under combined stress: under drought, excessive ethylene production promotes stress-induced senescence, and PGPR that degrade ACC maintain plants in a less senesced, more productive state during stress events. The comprehensive rice disease management review integrating metabolomics and transcriptomics for SynCom (synthetic community)-driven rhizomicrobiome engineering in salinity-stressed rice identifies enrichment in the hormone transduction gene category as one of the most consistent transcriptomic signatures of a PGPR-primed root microbiome versus an unprimed control (Castrillo et al., 2017; Sackey et al., 2025).

### 9.4 Microbiome engineering

Moving beyond inoculation of individual strains, microbiome engineering through the design of synthetic bacterial communities (SynComs) offers precision control over the collective hormone-modifying activity of the rhizosphere. SynComs are rationally designed consortia of selected strains whose hormone-production profiles are known and whose collective activity is predictable and complementary rather than competitive. SynCom-driven rhizomicrobiome engineering in salinity-stressed rice, supported by metabolomic and transcriptomic analyses, reveals mechanisms underlying plant adaptation to saline environments through the integration of multiple PGPR functions (Castrillo et al., 2017; Sackey et al., 2025). The key future challenge for field-scale SynCom deployment is stability: SynCom compositions optimized under controlled conditions frequently shift in competitive, microbiome-diverse field soil environments, particularly under variable temperature and moisture regimes. Developing SynCom formulations with strain combinations that maintain their defined hormone-production profiles under the specific abiotic-stress conditions they are designed to alleviate that is, SynComs for drought, different SynComs for combined drought-pathogen scenarios is the primary engineering target for the field (Castrillo et al., 2017; Sackey et al., 2025).

## 10 Emerging frontiers in hormone priming research

### 10.1 Epigenetic stress memory

Epigenetic stress memory, the heritable, non-sequence-based perpetuation of a primed chromatin state across cell divisions and potentially across generations represents the frontier where hormone priming and epigenomics converge. In rice, stress-induced epigenetic marks primed seeds are more resilient to drought, salinity, and temperature extremes in subsequent generations, potentially eliminating the need for repeated application of chemical priming agents (Dobránszki et al., 2025). CRISPR-dCas9 epigenome editing targeting H3K27me3, DNA methylation via DNMT3A fusion, and H3K4me3 enables precise modification of stress-related epigenetic marks and the development of crops with tailored stress responses. The ‘stress acclimation-epigenetic editing-stable trait inheritance’ paradigm proposed by (Cao and Chen, 2024; Song et al., 2025) represents the conceptual framework for converting transient hormone-priming effects into permanently engineered epigenetic states, a development that would transform phytohormone priming from an agronomic management practice into a stable genetic trait.

### 10.2 Biomolecular condensates

Biomolecular condensates, dynamic, non-membranous structures formed through liquid-liquid phase separation have emerged as an unexpected mechanism through which plant cells rapidly reorganize hormone-signaling components under stress without requiring transcriptional changes. Stress granules (SGs) and processing bodies (PBs), two major condensate types in rice, sequester specific mRNAs and signaling proteins under stress conditions, directly modulating which hormone-pathway transcripts are translated versus stored or degraded (Peng et al., 2025). The SG component TSN mediates SnRK1alpha condensation into SGs, promoting its heat-induced activation connecting biomolecular condensate formation directly to the ABA-pathway kinase activity that drives heat-stress hormone responses. In plant immunity, a surprisingly large number of fundamentally important cellular processes underlying JA- and SA-mediated defense are driven and regulated by biomolecular condensates, including condensation of key immune signaling components during pathogen attack (Chen et al., 2024). For rice hormone priming research, the implication is significant: priming stimuli may work partly by pre-configuring condensate composition poising specific mRNAs and kinases in condensates from which they can be rapidly mobilized rather than, or in addition to, altering gene expression or chromatin state.

### 10.3 Spatial hormone signaling

The spatial dimension of hormone signaling resolved by the spatial transcriptomics approaches described in Section 6.6 is now understood to be a fundamental organizing principle rather than a developmental detail. ABA originating from phloem tissues integrates local mechanical perception with whole-root adaptive responses (Zhong et al., 2025). SA and JA signaling during blast infection operate as tissue-wide broadcasts rather than cell-type-specific precision signals (Wang et al., 2025b). These spatial findings have direct implications for priming strategy design: if JA signaling is a uniform tissue-wide broadcast, then localized priming of specific cell types will not achieve targeted JA effects; whereas if ABA signals are spatially graded and phloem-derived, then vascular-specific delivery of ABA-pathway engineering will achieve disproportionate effects relative to constitutive approaches.

### 10.4 AI and systems biology

Artificial intelligence and systems biology approaches are transforming the translation of multi-omics hormone-network data into actionable engineering targets. Machine learning and deep learning algorithms efficiently process vast datasets from multi-omics experiments, enabling prediction of drug toxicity and gene function with high accuracy and accelerating identification of regulatory network hubs from data that would be intractable by conventional analysis (Dobránszki et al., 2025). For hormone-network specifically, WGCNA and graph neural network approaches are enabling identification of hormone-pathway hub genes from integrated rice multi-omics datasets identifying regulatory nodes whose expression predicts stress tolerance phenotypes across multiple stress combinations rather than just single stresses (Rajamuthu et al., 2025). The (Kang et al., 2025) Total Hormonal Sensitivity Index framework, presented in **Table 2**, represents the first quantitative AI-assisted mathematical modeling framework for ranking hormone-pathway responsiveness under combined stress, a direct predecessor of the predictive multi-omics models described in Section 10.6.

### 10.5 Digital twins of crops

A digital twin of a crop is a continuously updated computational model that simulates the crop’s physiological and molecular state in real time based on sensor inputs, genomic data, and environmental parameters enabling predictive rather than reactive management of stress events. For hormone priming applications, digital twin models that integrate the hormone-balance data described in this review could predict when a rice crop’s GA20/ABA ratio or JA-Ile/bioactive JA balance is trending toward stress-sensitivity and trigger targeted priming interventions before stress damage occurs. Machine learning models combining genomics, transcriptomics, hyperspectral imaging, and UAV-based phenotyping have moved from academic demonstrations to agricultural decision-support platforms for rice tracking yield prediction, precision irrigation scheduling, and pest detection simultaneously (Zhang et al., 2025). The integration of hormone-network models into these digital twin frameworks moving from phenotypic inputs to molecular mechanism inputs represents the next generation of the technology and is identified by the most comprehensive 2025 review of ML in agriculture as an emerging direction particularly promising for combined-stress scenarios (Zarbakhsh et al., 2025).

### 10.6 Predictive multi-omics models

The ultimate aspiration of the multi-omics, AI, and digital twin approaches described above is predictive multi-omics models that, given a rice genotype’s multi-omics profile under a defined stress condition, accurately predict its hormone-network response, its tolerance phenotype, and most valuably the minimal, highest-leverage genetic or chemical interventions that would optimize that response. (Wang et al., 2024 identify multi-omics integration frameworks combining network construction, machine learning, and big data visualization as the core technical infrastructure needed. For rice hormone priming specifically, the primary prediction target is the hormone balance not individual pathway activity levels, but the ratios described throughout this review as the mechanistically relevant variables: GA20/ABA, SA/JA, ABA/ethylene. Models that predict these ratios from early transcriptomic or epigenomic data before the stress phenotype is visible would enable priming interventions timed optimally to the plant’s current molecular state rather than to a standard calendar-based agricultural schedule.

## 11 Challenges and knowledge gaps

### 11.1 Complexity of hormone networks

The hormone crosstalk networks described in Section 5 are not merely complex they are context-dependent in ways that make systematic generalization genuinely difficult. The same SA-JA relationship that is antagonistic in the rice-Magnaporthe oryzae interaction is synergistic in the rice-virus interaction (Zhang et al., 2023a). The same ABA-JA synergism that is beneficial under combined drought-BPH stress becomes a resource-allocation problem when the two stresses are not co-occurring. The (Kang et al., 2025) Total Hormonal Sensitivity Index framework (**Table 2**) represents a promising attempt to quantify this context-dependence, but the framework has been validated only in a single pollutant-stress scenario. Extending such quantitative frameworks to the full combinatorial space of rice’s stress environment and validating them across diverse genotypic backgrounds remains the field’s most pressing mechanistic challenge.

### 11.2 Translation from laboratory to field

The vast majority of hormone priming and metabolic engineering results described in this review come from controlled environment studies growth chambers, hydroponic systems, greenhouse pots where stress timing, intensity, duration, and the absence of the full soil microbiome allow clean experimental designs but produce results that frequently fail to translate to open-field performance. The *OsPYL9* CRISPR knockout (**Table 3**) is notable precisely because it provides one of the few hormone-pathway engineering examples with validated field-condition yield improvement (Usman et al., 2020). The transition from controlled environments to field requires confronting highly variable temperatures and rainfall patterns, soil microbiome interactions, competing weed and pest pressures, and multi-year performance across diverse environments, all of which can erode the clean phenotypic advantages documented in controlled experiments.

To close the lab-to-field translation gap more systematically, we recommend the following concrete steps for future combined-stress field trials: (1) Multi-location evaluation of edited lines across sites with contrasting historical stress profiles (e.g., drought-prone lowland Asia, blast-endemic tropical environments) to capture genotype-by-environment interactions; (2) Use of multi-stress nurseries incorporating simultaneous pathogen inoculation under imposed water deficit, mimicking field conditions more faithfully than sequential single-stress experiments; (3) Integration with digital twin models and remote sensing platforms to enable real-time stress monitoring and adaptive management during field trials; (4) Adoption of multi-year, multi-season evaluation protocols to capture chronic combined-stress effects that short-season experiments miss; and (5) Collaboration with national agricultural research systems (NARS) in target countries to ensure that field trial environments are representative of the farmer-level stress scenarios that the edited cultivars are intended to address.

### 11.3 Multi-stress experimental design limitations

The field’s single most important methodological gap is the absence of standardized experimental frameworks for combined-stress studies in rice. Most published combined-stress studies test exactly two stresses simultaneously, under conditions defined by the researcher rather than by field-realistic stress profiles, and over short experimental windows that do not capture the accumulating effects of chronic combined stress across an entire growing season. Designing experiments that are simultaneously biologically realistic, statistically tractable, and comparable across research groups requires agreed upon stress combinations, intensities, developmental stage windows, and phenotyping outputs. The development of such standards analogous to the genomics community’s minimal information about a microarray experiment (MIAME) or the metabolomics community’s reporting standards is a practical priority for the combined-stress biology community.

### 11.4 Yield penalties

Despite the promising examples in this review where yield penalties have been dissociated from stress tolerance gains (*OsPYL9, OsJAZ10*), yield penalty remains the most common outcome of hormone-pathway engineering efforts. Constitutive overexpression of ABA-pathway genes typically delays germination, restricts vegetative growth under well-watered conditions, and reduces final grain yield even as it improves water-deficit survival. The GA-JA trade-off described in Section 5.5 similarly imposes a growth penalty that is structural it arises from the shared DELLA-JAZ corepressor system rather than from a technical limitation of current editing tools. Resolving yield penalties at scale requires moving from single-gene to multi-gene strategies that simultaneously edit the stress-tolerance-relevant component while compensating for its growth-restricting activity a target that multiplex editing and synthetic circuit approaches are positioned to address but have not yet achieved at field scale.

### 11.5 Regulatory challenges for genome-edited crops

The regulatory treatment of CRISPR-edited crops varies dramatically by jurisdiction in ways that directly limit the translation of the metabolic engineering strategies described in this review into deployable cultivars. The European Union’s recent regulatory update distinguishing between New Genomic Techniques (NGT1 and NGT2) creates a more permissive pathway for simple edits (NGT1) than for transgenic approaches, but most of the multiplex editing strategies described in Section 8 which simultaneously target three or four genes likely fall into the more heavily regulated NGT2 category. In countries where CRISPR-edited crops are treated equivalently to conventionally bred crops (including Argentina, the United States, and India), the commercial development path is substantially clearer. The regulatory divergence internationally means that varieties developed through multiplex hormone-pathway editing may face market access restrictions that limit their global deployment even where their performance is superior a non-biological but practically binding constraint that any strategic research roadmap for climate-resilient rice must explicitly acknowledge.

## 12 Future perspectives for sustainable agriculture

(This section directly addresses the Special Issue theme: ‘Future perspectives and implications for sustainable agriculture’).

### 12.1 Next-generation metabolic engineering

The metabolic engineering strategies described in Section 7 are currently limited by their reliance on relatively simple regulatory architectures constitutive or stress-inducible promoters driving single hormone-pathway genes. Next-generation metabolic engineering will combine multiplex CRISPR editing of hormone-network hubs, stress-sensing synthetic gene circuits that respond to hormone signals as their inputs, and epigenome editing to establish stable, heritable chromatin states at key regulatory loci. The target is a rice plant whose hormone balance under combined stress is architecturally stable not elevated constitutively in a way that imposes yield penalties, but programmed to shift rapidly and precisely toward the stress-appropriate balance point when stress signals are detected, then reset to the growth-optimized balance when stress subsides. This kind of dynamic, reversible hormone-balance programming is achievable in principle with the synthetic biology tools now available (Lloyd et al., 2025) but has not yet been demonstrated in rice under field conditions.

### 12.2 Precision breeding

Precision breeding integrates genomic selection, CRISPR editing, and multi-omics phenotyping into a single accelerated breeding cycle that can install targeted genetic changes in elite cultivar backgrounds without the multi-year backcrossing cycles of conventional introgression. For hormone-priming-relevant traits, precision breeding will exploit the quantitative hormonal sensitivity framework (Kang et al., 2025) to identify the genotypic combinations that produce optimal hormone balance under combined stress, then use CRISPR to install those combinations in elite backgrounds. Marker-assisted selection for epialleles heritable DNA methylation differences at hormone-responsive loci that are associated with tolerance class without requiring sequence changes represents a complementary low-regulatory-burden precision breeding approach that is currently feasible in rice given the methylome data available for diverse cultivars (Garg et al., 2015). The combination of sequence-level CRISPR editing and methylome-level epiallele selection in a single breeding program is a realistic near-term target that has not yet been systematically attempted. In summary, the convergence of mechanistic understanding of phytohormone crosstalk, multi-omics-guided target discovery, precision genome editing, microbiome-assisted delivery, and AI-enabled predictive modeling has created an unprecedented opportunity to develop rice cultivars that are not merely tolerant of individual stresses but genuinely resilient to the unpredictable combinatorial stress landscape of climate change. The scientific and technological foundations described in this review are now sufficiently mature to support integrated, field-directed research programs; capitalizing on this convergence before escalating climate volatility outpaces agricultural adaptation represents one of the most consequential priorities in contemporary crop science.

### 12.3 Pathways to commercialization and adoption

Translating the hormone priming and metabolic engineering strategies reviewed here into deployed agricultural technologies requires explicit attention to economic feasibility, adoption barriers, and regulatory pathways. Chemical phytohormone priming (ABA, JA, SA foliar sprays) offers the lowest per-hectare cost but requires repeated seasonal application; cost-benefit analyses in smallholder rice systems suggest that priming chemicals must achieve >15% yield protection to be economically viable at current commodity prices. PGPR and SynCom-based priming formulations offer a more durable alternative with lower per-season costs once optimized, and several Bacillus-based bioformulations have already achieved commercial registration in Asia. CRISPR-edited varieties, including the Sub1A submergence-tolerant lines already deployed at scale across Bangladesh, India, and the Philippines, demonstrate that genome-edited or transgene-free edited crops can be released within regulatory frameworks that treat them equivalently to conventionally bred varieties in countries including India, the Philippines, the United States, and Argentina. For smallholder adoption, the critical bottleneck is seed system integration: edited seed must be available through established public seed supply chains or licensed to commercial breeders. Regulatory and intellectual property considerations are increasingly being addressed through open-licensing frameworks (e.g., IRRI’s open-access licensing for Sub1 alleles) and government-backed trait licensing models. The commercialization trajectory for the multiplex-editing strategies described in this review will depend critically on whether target trait packages can be validated in elite, locally adapted cultivar backgrounds within 5–7-year regulatory timelines.

### 12.4 Climate-smart rice development

Climate-smart rice development reframes the breeding objective from ‘tolerance to a defined stress’ to ‘agronomic performance stability across a defined range of future climate scenarios.’ Under this reframing, the optimal variety is not the one that performs best under drought or best under heat, but the one whose hormone-network architecture produces the most consistent performance across the full combinatorial space of stresses predicted under CMIP6 scenarios for the target environment. This requires explicit integration of climate projection data into variety development pipeline design identifying which stress combinations will be most prevalent in a target region under 2050 climate scenarios, then engineering hormone-network architectures specifically robust to those combinations rather than to historical stress profiles. The digital twin and predictive multi-omics model approaches described in Section 10 are the methodological infrastructure through which this climate projection integration is achievable.

### 12.5 Integrating multi-omics and AI

The convergence of multi-omics data streams transcriptomics, proteomics, metabolomics, epigenomics, and spatial omics with AI-based network analysis will progressively resolve the hormone-network architecture of rice stress adaptation to a resolution that enables rational engineering rather than hypothesis-driven single-gene studies. The near-term priority is developing AI models trained on the integrated multi-omics datasets now becoming available (**Table 2** represents the current state; the datasets underlying those studies contain far more information than the single-sentence findings captured in the table). These models, trained on hormone-balance data across diverse stress conditions and genotypic backgrounds, will generate ranked lists of engineering targets specific genes, specific epigenetic marks, specific SynCom compositions whose modification is predicted to optimize hormone balance for a defined combined-stress scenario. Validation of these AI-generated targets through multiplexed CRISPR editing in controlled environments, followed by rapid advancement to field trials, defines the integrated research pipeline that the field is moving toward.

### 12.6 Towards resilient rice cultivars

**Figure 4:**
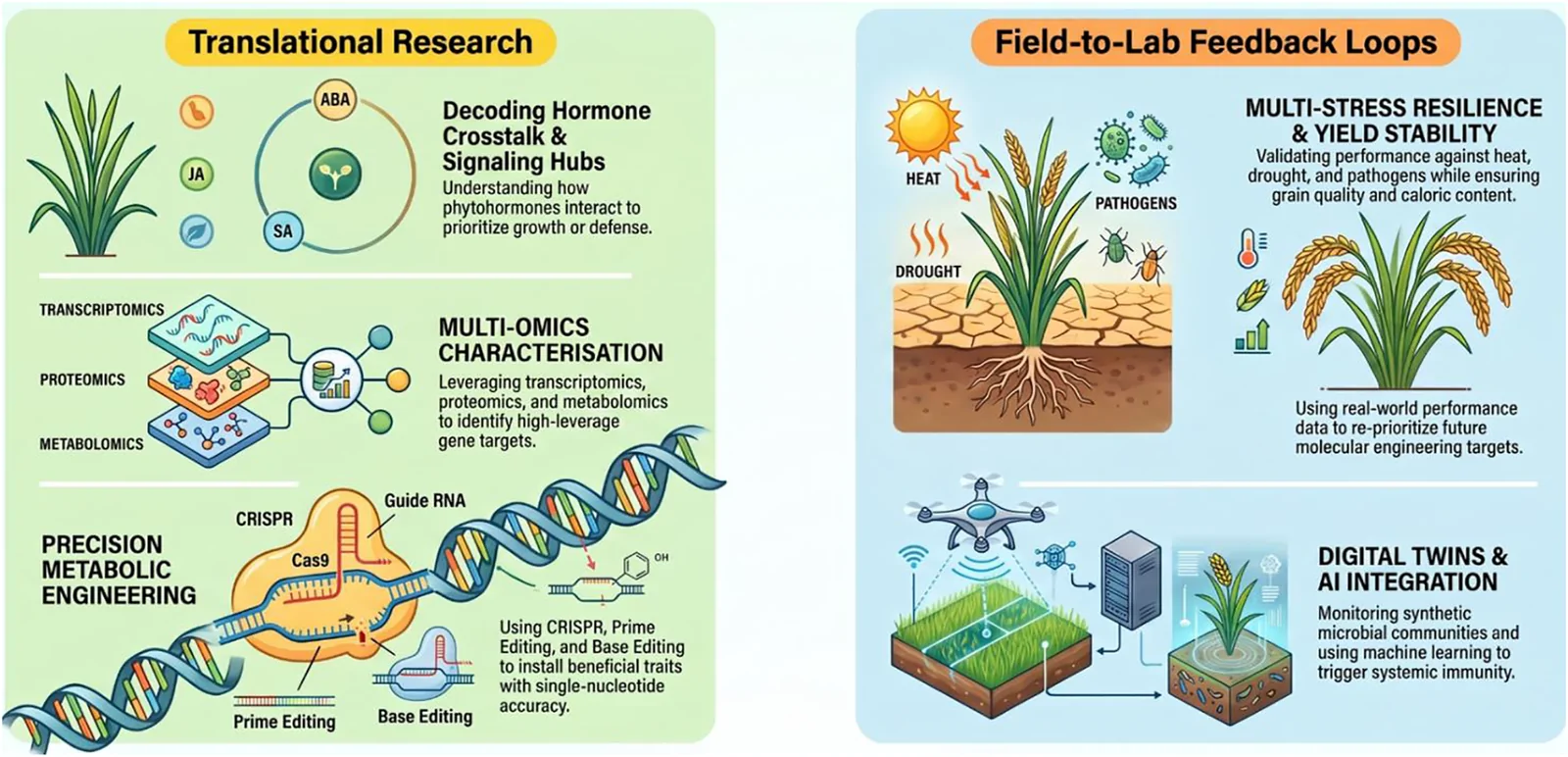
Integrated research pipeline for developing multi-stress resilient cultivars.

The ultimate objective of the research synthesized in this review is the development of rice cultivars that maintain acceptable yield and grain quality across the full range of biotic and abiotic stresses predicted under climate change resilient cultivars in the true sense, not just tolerant cultivars under any single stress. The pathway to such cultivars runs through the convergence of the five research streams described in this review: (1) mechanistic understanding of hormone-network crosstalk under combined stress (Sections 4-5); (2) multi-omics resolution of the molecular architecture of priming and stress memory (Section 6 and **Table 2**); (3) metabolic engineering and CRISPR precision editing of hormone pathway and crosstalk nodes (Sections 7–8 and **Table 3**); (4) microbiome-assisted priming as a field-deployable complementary strategy (Section 9); and (5) AI and digital twin integration for real-time, variety-specific priming guidance (Section 10). None of these streams alone is sufficient. Their integration, guided by the principle that hormone balance under combined stress is the key phenotypic variable, and executed through the combined application of precision editing and microbiome engineering, represents the most tractable current pathway to the climate-resilient rice varieties that global food security under climate change demands (**Figure 4**).

This schematic illustrates the transition from basic biological discovery to the development of field-ready, stress-resilient crops. Fundamental Science (Bottom Tier): Focuses on a mechanistic understanding of hormone crosstalk. It involves decoding how major phytohormones (such as ABA, JA, and SA) interact to balance growth versus defense. Key signaling hubs, particularly transcription factor families like *WRKY, MYB*, and *NAC*, are identified as network arbitrators for multiple stress inputs. A critical component is resolving the DELLA-JAZ co-repressor interface to decouple plant immunity from yield loss. Translational Research (Middle Tier): Utilizes multi-omics characterization (transcriptomics, proteomics, and metabolomics) combined with AI and Machine Learning to identify high-leverage gene targets. Precision metabolic engineering is achieved through advanced tools like CRISPR, Prime Editing (PE), Base Editing (BE), and Epigenome editing to install beneficial traits with single-nucleotide accuracy. This tier also includes the design of Synthetic Signaling Circuits using AND-gate logic to activate stress-response genes only under specific environmental triggers. Field Application (Top Tier): Represents the final stage of field-validated cultivar development. Edited lines (e.g., rice) are grown in diverse, complex environments to test resilience against heat, drought, and pathogens. Technologies such as Digital Twins (DT) and SynCom microbiome-assisted priming are used to monitor and trigger systemic immunity in the field while ensuring Yield & Quality Stability maintaining grain milling quality and caloric content for food security. Field-to-Lab Feedback Loops: Data from real-world field performance is continuously fed back into the pipeline to re-priorities future molecular engineering targets.

## 13 Conclusions

This review has synthesized current understanding of phytohormone priming and crosstalk in rice under combined biotic and abiotic stresses, integrating mechanistic biology with multi-omics advances and engineering strategies to address the most pressing challenge in rice research: developing varieties capable of maintaining yield and quality across the combinatorial stress landscape of climate change. Five overarching conclusions emerge:

First, phytohormone priming is a powerful and underutilized strategy for stress resilience. Pre-exposure of rice to mild hormone elicitors whether chemical, microbial, or physical consistently improves subsequent stress responses by lowering the activation threshold of multiple defense pathways simultaneously. The diversity of priming methods now available (hydropriming, osmopriming, hormonal priming, bio-priming, nanoparticle delivery, SynCom inoculation) and the mechanistic understanding of the chromatin marks and signaling poising that underlie priming effects provide a strong scientific foundation for developing priming-based agronomic protocols that are practical at field scale.

Second, hormonal crosstalk determines rice adaptation under combined stresses. The pairwise interactions described in Section 5 ABA-JA synergism, ABA-SA antagonism, JA-SA conditional synergism and antagonism, ethylene’s context-setting role, and the structural growth-defense trade-off running through the DELLA-JAZ co-repressor system collectively mean that the outcome of any single-hormone manipulation under combined stress is inherently unpredictable without knowledge of the hormonal network state. The convergence-onto-shared-hubs model (*WRKY, MYB,* MYC2, *NAC*, and *bZIP* transcription factors as network arbitrators) provides a unifying framework for understanding these interactions and a rational basis for selecting engineering targets that shift network balance rather than individual pathway outputs.

Third, multi-omics platforms are uncovering previously hidden regulatory networks. The nine studies in **Table 2** collectively demonstrate that no single omics layer not transcriptomics, not proteomics, not metabolomics can generate the complete mechanistic picture of hormone priming that combined-stress tolerance engineering requires. Hormone balance (GA20/ABA ratios, JA-Ile/bioactive JA ratios, SA/JA ratios) is only measurable through targeted hormonomics; the epigenetic marks that sustain stress memory are only detectable through WGBS and histone ChIP-seq; the cell-type specificity of stress responses is only resolvable through single-cell and spatial transcriptomics. The integrated multi-omics datasets now being generated across diverse rice genotypes, stress combinations, and developmental stages constitute the most valuable scientific resource available for identifying the hormone-network architecture of combined-stress tolerance.

Fourth, metabolic engineering and genome editing provide practical tools for translating mechanistic insights into crop improvement. The six CRISPR-edited genes in **Table 3** demonstrate that hormone-pathway engineering can now produce: yield improvement with drought tolerance (*OsPYL9*); broad-spectrum disease resistance (OsS5H1/2/3); insect resistance without growth penalty (*OsJAZ10*); and direct evidence of JA-ABA crosstalk mechanistic coupling (*OsJAZ12/13*). Base editing and prime editing extend this capability to single-nucleotide precision and all 12 transition types without double-strand breaks, while multiplex editing enables simultaneous targeting of hormone-network hubs opening the realistic near-term prospect of varieties whose hormone balance is comprehensively optimized for combined-stress tolerance.

Fifth, the integration of hormone priming, multi-omics characterization, metabolic engineering, microbiome-assisted delivery, and AI-guided predictive modeling will accelerate the development of climate-resilient rice varieties on a timeline commensurate with the rate of climate change. No single approach is sufficient; climate resilience in rice is a systems property that will require systems-level solutions. The research roadmap proposed in Section 12 mechanistic understanding of hormone crosstalk under combined stress, multi-omics resolution of the architecture of that crosstalk, precision editing of the identified crosstalk nodes, microbiome-assisted priming for field-scale deployment, and AI-digital twin integration for real-time management provides the integrated framework through which this goal is achievable.
